# Metabolite Profiling Reveals Distinct Modulation of Complex Metabolic Networks in Non-Pigmented, Black, and Red Rice (*Oryza sativa* L.) Cultivars

**DOI:** 10.3390/metabo11060367

**Published:** 2021-06-09

**Authors:** Tae Jin Kim, So Yeon Kim, Young Jin Park, Sun-Hyung Lim, Sun-Hwa Ha, Sang Un Park, Bumkyu Lee, Jae Kwang Kim

**Affiliations:** 1 Division of Life Sciences, College of Life Sciences and Bioengineering, Incheon National University, Incheon 22012, Korea; f91gd@inu.ac.kr (T.J.K.); aliana79@inu.ac.kr (S.Y.K.); pyj2050@inu.ac.kr (Y.J.P.); 2 Division of Horticultural Biotechnology, School of Biotechnology, Hankyong National University, Anseong 17579, Korea; limsh2@hknu.ac.kr; 3 Department of Genetic Engineering and Graduate School of Biotechnology, Kyung Hee University, Yongin 17104, Korea; sunhwa@khu.ac.kr; 4 Department of Crop Science, Chungnam National University, 99 Daehak-ro, Yuseong-gu, Daejeon 34134, Korea; supark@cnu.ac.kr; 5 Department of Environment Science & Biotechnology, Jeonju University, Jeonju 55069, Korea

**Keywords:** carbon, metabolomics, metabolite profiling, multivariate analysis, nitrogen, PathVisio 3, pigmented rice, terpenoid

## Abstract

Comprehensive profiling of primary and secondary metabolites was performed to understand metabolic differences associated with color formation in pigmented rice (*Oryza sativa* L.). Overall, 110 metabolites from non-pigmented, black, and red rice cultivars were identified. Black and red rice contained high levels of flavonoids associated with plant color. Black rice also contained high levels of terpenoids (carotenoids, tocopherols, phytosterols, and monoterpenes). The non-pigmented rice contained relatively low levels of secondary metabolites. Multivariate and pathway analyses were performed to data-mine the metabolite profiles. Hierarchical clustering analysis of correlation coefficients revealed metabolite clusters based on nitrogen and carbon sources. These clusters suggested a negative correlation between nitrogen and carbon. Pathway analysis revealed that black rice was rich in carbon-based secondary metabolites, with relatively low levels of primary metabolites compared with other rice cultivars. These data highlight the complex interactions between nitrogen and carbon metabolism of primary and secondary metabolites in rice. For the first time, the relationships and metabolic differences in terpenoid content (monoterpenes, triterpenes, and tetraterpenes) of non-pigmented and pigmented rice cultivars were analyzed. These findings should greatly contribute to the understanding of pigmented rice metabolome and inform breeding programs for new rice cultivars.

## 1. Introduction

Rice (*Oryza sativa* L.) is a staple diet for over half of the world’s population and is the second largest cultivated cereal crop worldwide [1,2]. Pigmented rice comes in various colors, such as black, red, and green. The major coloring agents in black rice (BR) are anthocyanins, such as cyanidin-3-*O*-glucoside and peonidin-3-*O*-glucoside, whereas those in red rice (RR) are proanthocyanidins and flavan-3-ols oligomers, with catechin as the main synthesis unit [3,4]. Consumption of pigmented rice is associated with several health benefits, for example, antioxidant, anti-cancer, anti-tumor, anti-diabetic, and cardiovascular-protective activities [5].

Genotypic diversity of phytochemicals linked to the rice color has been described in several recent studies on pigmented rice cultivars [6,7,8]. However, to date, most of the research into pigmented rice has focused on the relationship between anthocyanins and antioxidants, and on nutrients, because rice is considered to be a functional food in many Asian countries, with several health-promoting effects reported [9]. For example, BR contains high levels of anthocyanins, carotenoids, phytosterols, tocopherols, protocatechuic acid, and other phenolics [3,10,11], whereas RR contains high levels of proanthocyanidins and other phenolics [12,13]. Furthermore, the antioxidant activity of pigmented rice is higher than that of non-pigmented rice. The phenolic and flavonoid contents, and antioxidant activity are significantly and positively correlated [11,14].

The pleasant aroma of rice during or after cooking is an important preference factor for the consumer [15]. This pleasant odor has been extensively studied. The major volatile compound of raw and cooked rice is 2-acetyl-1-pyrroline (responsible for popcorn aroma) [9]. BR varieties with a relatively strong aroma have been identified, and contain more 2-acetyl-1-pyrroline, guaiacol, indole, and *p*-xylene than white rice (WR) [16]. Sukhonthrea et al. [9] compared the volatile aroma compounds of BR and RR. The main volatiles of RR were identified as myristic acid, nonanal, (*E*)-β-ocimene, and 6,10,14-trimethyl-2-pentadenone. In BR, these were myristic acid, nonanal, caproic acid, pentadecanal, and pelargonic acid.

To date, only few metabolomic studies on pigmented and non-pigmented rice have been published. In one such study, Kim et al. [3] reported that the levels of flavonoids and carotenoids in pigmented rice are positively correlated. Furthermore, Kim et al. [11] showed that the levels of all phenolics and shikimic acid are positively correlated in BR and WR. In another study, Kim et al. [17] observed that phenolic acid composition of rice grain is determined by environmental factors rather than genetic factors. Furthermore, Liu et al. [18] presented metabolic explanation for the yellowing mechanism during rice storage using non-targeted metabolomics. However, to date, no reports on the correlations between monoterpenes, sesquiterpenes, triterpenes, and tetraterpenes in non-pigmented and pigmented rice have been published.

The various metabolites present in pigmented rice have been studied in detail. However, to the best of our knowledge, comprehensive metabolic profiling, including that of primary and secondary metabolites in various pigmented rice cultivars is lacking, as is the understanding of metabolic networks linking big data from metabolite profiling with metabolic pathways.

In this study, we present a comprehensive interpretation of metabolic networks in rice. Non-pigmented (WR), red-pigmented (RR), and black-pigmented (BR) rice were analyzed. We performed extensive metabolite profiling of primary and secondary metabolites, specifically, organic acids, sugars, sugar alcohols, amino acids, phenolic acids, flavan-3-ols (catechin and epicatechin), anthocyanins (peonidin-3-*O*-glucoside and cyanidin-3-*O*-glucoside), tocopherols, carotenoids, phytosterols, fatty acids, and volatiles) by using gas chromatography-quadrupole mass spectrometry (GC-qMS), GC×GC-time-of-flight (TOF) mass spectrometry (MS), GC-flame ionization detection (FID), head-space (HS)-GC-TOF-MS, high-performance liquid chromatography (HPLC), and LC-MS. To interpret the generated big data to allow understanding of metabolic differences between the rice samples, multivariate statistical analyses (principal component analysis, PCA; partial least squares discriminant analysis, PLS-DA; orthogonal partial least squares discriminant analysis, OPLS-DA; Pearson’s correlation analysis; and hierarchical clustering analysis, HCA), and a tool for visualization and analysis of metabolic pathways (PathVisio 3.3.0) were used. The study considerably broadens the understanding of rice metabolome in different rice cultivars, and may be used to inform the breeding of new cultivars.

## 2. Results and Discussion

### 2.1. PCA, PLS-DA, and OPLS-DA

To elucidate the overall metabolome patterns in rice seeds of three different colors, GC×GC-TOF-MS, solid-phase micro-extraction (SPME)-GC-TOF-MS, GC-qMS, GC-FID, HPLC-MS, and HPLC-UV were used to comprehensively profile primary and secondary metabolites in seeds of 16 cultivars of rice. Overall, 110 metabolites, including amino acids, organic acids, sugars, sugar alcohols, phenolic acids, flavonoids, anthocyanins, carotenoids, phytosterols, policosanols, tocopherols, fatty acids, and volatiles, were identified. 

Multivariate statistical analysis is an important tool used for obtaining an overview of patterns in complex experimental data. Specifically, PCA is a preliminary step in a multivariate analysis performed to discern novel information hidden in the big data. Accordingly, PCA of the obtained metabolite profiles was performed so that each point in the score plot indicated an individual sample, and samples with similar metabolite composition clustered together. PCA did not reveal a clear separation of the rice seeds by color (Figure S1).

**Figure 1:**
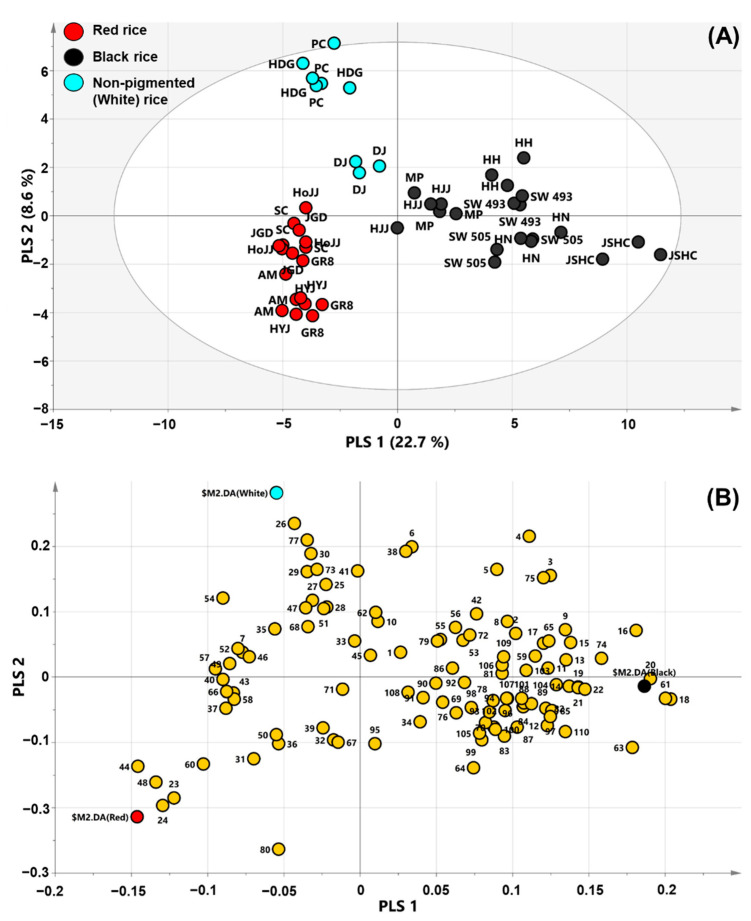
Partial least squares-discriminant analysis (PLS-DA) score (**A**) and loading plots (**B**) derived from 110 metabolites of black, red, and non-pigmented (white) rice cultivars. The ellipse represents the Hotelling T2 with 95% confidence in the score plot. Plot annotation 1, C20-ol (Eicosanol); 2, C21-ol (Heneicosanol); 3, C22-ol (Docosanol); 4, C24-ol (Tetracosanol); 5, C26-ol (Hexacosanol); 6, β-Tocopherol; 7, γ-Tocopherol; 8, C27-ol (Heptacosanol); 9, C28-ol (Octacosanol); 10, γ-Tocotrienol; 11, α-Tocopherol; 12, Cholesterol; 13, α-Tocotrienol; 14, Campesterol; 15, C30-ol (Triacontanol); 16, Stigmasterol; 17, β-Sitosterol; 18, Lutein; 19, Zeaxanthin; 20, β-Carotene; 21, Cyanidin-3-*O*-glucoside; 22, Peonidin-3-*O*-glucoside; 23, Catechin; 24, Epicatechin; 25, C16:0 (Palmitic acid); 26, C18:0 (Stearic acid); 27, C18:1 (Oleic acid); 28, C18:2 (Linoleic acid); 29, C18:3 (α-Linolenic acid); 30, C20:0 (Arachidonic acid); 31, Pyruvic acid; 32, Lactic acid; 33, Alanine; 34, Oxalic acid; 35, Valine; 36, Ethanolamine; 37, Leucine; 38, Glycerol; 39, Phosphoric acid; 40, Isoleucine; 41, Proline; 42, Glycine; 43, Succinic acid; 44, Fumaric acid; 45, Serine; 46, Threonine; 47, β-Alanine; 48, Malic acid; 49, Salicylic acid; 50, Methionine; 51, Aspartic acid; 52, Pyroglutamic acid; 53, γ-Aminobutyric acid; 54, Cysteine; 55, Threonic acid; 56, Glutamic acid; 57, *p*-Hydroxy benzoic acid; 58, Phenylalanine; 59, Asparagine; 60, Xylose; 61, Vanillic acid; 62, Glutamine; 63, Protocatechuic acid; 64, Shikimic acid; 65, Citric acid; 66, Fructose; 67, Galactose; 68, Lysine; 69, Glucose; 70, *p*-Coumaric acid; 71, Tyrosine; 72, Mannitol; 73, Ferulic acid; 74, Inositol; 75, Caffeic acid; 76, Tryptophan; 77, Sinapinic acid; 78, Sucrose; 79, Raffinose 80, 1-Butanol; 81, Pentanal; 82, 1-Pentanol; 83, Toluene; 84, Hexanal; 85, 1-Hexanol; 86, Ethylbenzene; 87, *p*-Xylene; 88, 2-Heptanone; 89, 2-Butyl furan; 90, Styrene; 91, Heptanal; 92, 2-Acetyl-1-pyrroline; 93, α-Pinene; 94, 1-Heptanol; 95, Benzaldehyde; 96, 1-Octen-3-ol; 97, 2-Pentyl furan; 98, Octanal; 99, *p*-Cymene; 100, D-Limonene; 101, 3-Octen-2-one; 102, γ-Terpinene; 103, 1-Octanol; 104, Linalool; 105, o-Cymene; 106, Nonanal; 107, 1-Nonanol; 108, Naphthalene; 109, Decanal; 110, 1H-Indole.

PLS-DA was then performed to optimize the separation of samples and to determine metabolic differences arising from the rice color. PLS-DA is a projection method, which rotates the PCA projection to obtain maximum separation by classes of observations based on their variables. In the analysis, the rice seed colors were set as classes and an internal validation method was used for model validation. For the latter, validation parameters (*R*^2^ and *Q*^2^) indicate the quality of the model. *R*^2^ specifies the proportion of variation in the data that is explained by the model, and *Q*^2^ specifies the proportion of variation in the data that is predictable by the model. The *R*^2^- and *Q*^2^-values fall between zero and one: *R^2^* close to 1 is desirable; *Q*^2^ > 0.5 indicates a good prediction model; and *Q*^2^ > 0.9 indicates an excellent prediction model. PLS-DA showed clear separation by color of rice seeds (Figure 1A). The validation analysis of the PLS-DA model yielded *R*^2^X of 0.312, *R*^2^Y of 0.792, and *Q*^2^ of 0.725. The *Q*^2^-value was larger than 0.50, indicating a good predictive ability of the model. PLS 1 significantly contributed to the separation of BR from other rice samples. Metabolites in the loading plots explain the separation of corresponding samples on score plots. Significant metabolites of PLS 1 were fumaric acid, malic acid, epicatechin, catechin, xylose, lutein, vanillic acid, β-carotene, stigmasterol, and protocatechuic acid, for which the eigenvector values were −0.145934, −0.133728, −0.129379, −0.122005, −0.103077, 0.203814, 0.200285, 0.190348, 0.180684, and 0.178313, respectively (Figure 1B and Table S1). The eigenvector values of fumaric acid, malic acid, epicatechin, catechin, and xylose were negative, and contributed to the separation of BR from RR, and their content in RR was higher than that in other rice samples (Tables S2 and S3). RR contains high amounts of proanthocyanidin, which is composed of oligomers of catechin and epicatechin, and has a red color [4,19]. Hence, catechin and epicatechin significantly contributed to the separation of RR from other rice samples. By contrast, lutein, vanillic acid, β-carotene, stigmasterol, and protocatechuic acid, which had positive eigenvector values, contributed significantly to BR identity, and their amounts were higher in BR than in RR and WR (Tables S1, S2 and S4). According to a previous study, BR contains high amounts of carotenoids and phytosterols [3,20]. This is in agreement with our findings. Stigmasterol was the most abundant phytosterol in BR (Table S4). The BR color is driven by anthocyanins, such as peonidin-3-glucoside and cyanidin-3-glucoside [21,22]. Furthermore, most volatiles, such as, nonanal, octanal, benzaldehyde, 2-heptanone, 1-octen-3-ol, and naphthalene, were more abundant in BR than in other rice samples (Table S5). In addition, inositol was also identified as an important contributor to the separation of BR from others in the PLS-DA loading plots (Table S1). Based on a previous study, BR contains more inositol than RR and WR [23]. These previously reported observations were consistent with our findings in the present study (Table S2).

The important metabolites of PLS 2 were C18:0, sinapinic acid, C20:0, ferulic acid, β-tocopherol, 1-butanol, catechin, epicatechin, malic acid, and shikimic acid, for which the eigenvector values were 0.234828, 0.209490, 0.189271, 0.164575, 0.200056, −0.26382, −0.19669, −0.18505, −0.16048, and −0.13863, respectively (Table S1). These metabolites significantly contributed to the separation of non-pigmented rice (WR) from pigmented rice. In addition, the analysis revealed that WR contained more fatty acids than other rice samples.

**Figure 2:**
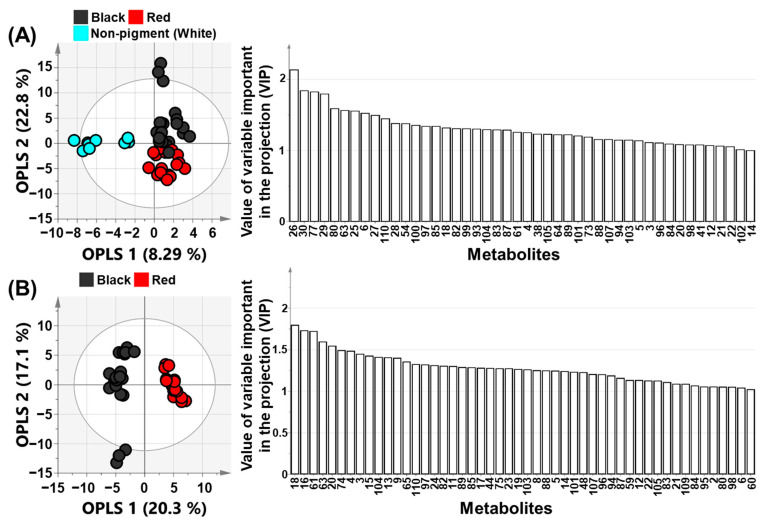
Orthogonal partial least squares-discriminant analysis (OPLS-DA) score and variable importance in the projection (VIP) plots derived from 110 metabolites of black, red, and non-pigmented (white) rice cultivars (**A**,**B**). A is comparison of non-pigmented rice and pigmented rice. B is comparison of black and red pigmented rice. The ellipse represents the Hotelling T2 with 95% confidence in the score plot. Plot annotation 1, C20-ol (Eicosanol); 2, C21-ol (Heneicosanol); 3, C22-ol (Docosanol); 4, C24-ol (Tetracosanol); 5, C26-ol (Hexacosanol); 6, β-Tocopherol; 7, γ-Tocopherol; 8, C27-ol (Heptacosanol); 9, C28-ol (Octacosanol); 10, γ-Tocotrienol; 11, α-Tocopherol; 12, Cholesterol; 13, α-Tocotrienol; 14, Campesterol; 15, C30-ol (Triacontanol); 16, Stigmasterol; 17, β-Sitosterol; 18, Lutein; 19, Zeaxanthin; 20, β-Carotene; 21, Cyanidin-3-*O*-glucoside; 22, Peonidin-3-*O*-glucoside; 23, Catechin; 24, Epicatechin; 25, C16:0 (Palmitic acid); 26, C18:0 (Stearic acid); 27, C18:1 (Oleic acid); 28, C18:2 (Linoleic acid); 29, C18:3 (α-Linolenic acid); 30, C20:0 (Arachidonic acid); 31, Pyruvic acid; 32, Lactic acid; 33, Alanine; 34, Oxalic acid; 35, Valine; 36, Ethanolamine; 37, Leucine; 38, Glycerol; 39, Phosphoric acid; 40, Isoleucine; 41, Proline; 42, Glycine; 43, Succinic acid; 44, Fumaric acid; 45, Serine; 46, Threonine; 47, β-Alanine; 48, Malic acid; 49, Salicylic acid; 50, Methionine; 51, Aspartic acid; 52, Pyroglutamic acid; 53, γ-Aminobutyric acid; 54, Cysteine; 55, Threonic acid; 56, Glutamic acid; 57, *p*-Hydroxy benzoic acid; 58, Phenylalanine; 59, Asparagine; 60, Xylose; 61, Vanillic acid; 62, Glutamine; 63, Protocatechuic acid; 64, Shikimic acid; 65, Citric acid; 66, Fructose; 67, Galactose; 68, Lysine; 69, Glucose; 70, *p*-Coumaric acid; 71, Tyrosine; 72, Mannitol; 73, Ferulic acid; 74, Inositol; 75, Caffeic acid; 76, Tryptophan; 77, Sinapinic acid; 78, Sucrose; 79, Raffinose 80, 1-Butanol; 81, Pentanal; 82, 1-Pentanol; 83, Toluene; 84, Hexanal; 85, 1-Hexanol; 86, Ethylbenzene; 87, *p*-Xylene; 88, 2-Heptanone; 89, 2-Butyl furan; 90, Styrene; 91, Heptanal; 92, 2-Acetyl-1-pyrroline; 93, α-Pinene; 94, 1-Heptanol; 95, Benzaldehyde; 96, 1-Octen-3-ol; 97, 2-Pentyl furan; 98, Octanal; 99, *p*-Cymene; 100, D-Limonene; 101, 3-Octen-2-one; 102, γ-Terpinene; 103, 1-Octanol; 104, Linalool; 105, o-Cymene; 106, Nonanal; 107, 1-Nonanol; 108, Naphthalene; 109, Decanal; 110, 1H-Indole.

OPLS-DA was next performed to delineate the differences in metabolism in rice samples by maximum separation (Figure 2). OPLS-DA is an extension of a supervised PLS method. In this approach, the X-variables separate the systematic variation into two parts, one that models the correlation between X and Y (prediction), and one that models the orthogonal components. Thus, OPLS-DA yields maximum separation by classes of observations based on their variables. Consequently, OPLS-DA outcomes are more easily interpreted than PLS-DA outcomes. At first, data for non-pigmented (WR) and pigmented rice were compared to construct the OPLS-DA model for identifying the metabolic differences determined by the formation of rice pigment. Non-pigmented rice (WR) and pigmented rice groups were compared in OPLS-DA models (Figure 2A). The Y-variables for WR were set to 0 and those for pigmented rice to 1. The score plots of OPLS-DA model showed good separation. The projection model of WR and pigmented rice group showed *R*^2^X of 0.311, *R*^2^Y of 0.823, and *Q*^2^ of 0.753. The *Q*^2^-value was higher than 0.50, indicating a good prediction power of the model. Two OPLS in the score plots explained 31.1% of the total variance (OPLS 1, 8.29%; OPLS 2, 22.8%). OPLS 1 explained the separation of WR and pigmented rice, whereas OPLS 2 elucidated the separation of RR and BR. Variable importance in projection (VIP) plots were used to explain the contribution of metabolites to the OPLS models. VIP values greater than 1.00 indicate a significant influence on the model. Overall, 46 metabolites in the VIP plot had VIP values greater than 1.00 (Table S6). In the analysis, C18:0, C20:0, sinapinic acid, and C18:3 were top-ranked metabolites. These results were in agreement with PLS-DA data. However, OPLS 2 had a greater explanatory power in this OPLS-DA model than OPLS 1. Next, OPLS-DA was performed for BR and RR groups to clarify the metabolic differences between them (Figure 2B). The validation parameters of the projection model were *R*^2^X of 0.374, *R*^2^Y of 0.986, and *Q*^2^ of 0.935. The *Q*^2^-value was above 0.90, indicating excellent prediction ability of this model. In addition, the score plot showed good separation by color. Overall, 50 metabolites in VIP plots had cut-off values above 1.00 (Table S7). Lutein, stigmasterol, vanillic acid, protocatechuic acid, and β-carotene were top-ranked metabolites in VIP plots. These results were consistent with those of PLS-DA. 

Finally, the two OPLS-DA models were tested by a permutation test and analysis of variance of the cross-validated residuals (CV-ANOVA) to determine the risk of over-fitting the OPLS model (Figure S2). The permutation test was performed with 200 permuted models generated by using randomized Y-variables. When the *Q*^2^-value of the permutation test is smaller than that of the actual (unpermuted) OPLS model, the model is considered to be predictive. Both OPLS models had *Q*^2^-values smaller than those of the permuted models. Hence, the two OPLS-DA models were predictive. Furthermore, *p*-value in the CV-ANOVA test of the two OPLS models was lower than 0.05 (pigmented rice vs. non-pigmented rice (WR), 1.48 × 10^−15^; BR vs. RR, 1.20 × 10^−21^). A *p*-value below 0.05 indicates that the model is validated.

### 2.2. Pearson’s Correlation Analysis and HCA

**Figure 3:**
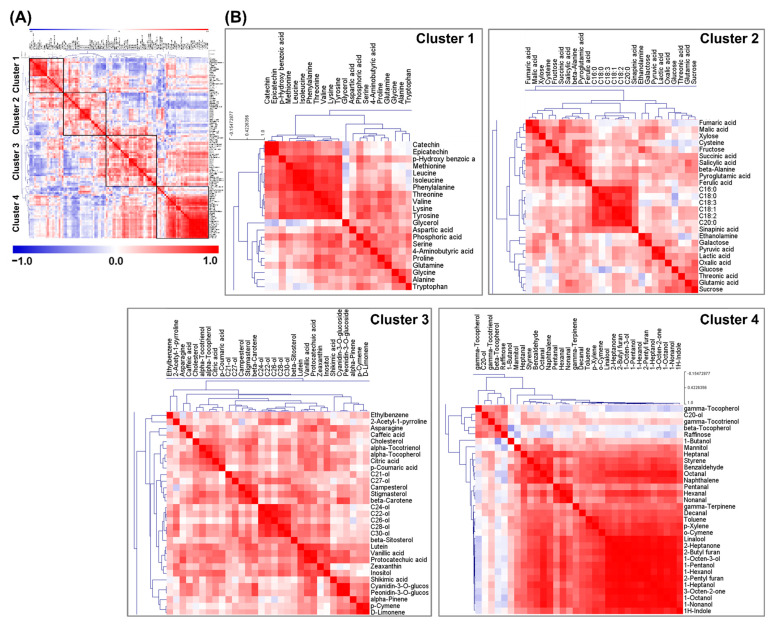
Correlation matrix and cluster analysis of data for 110 metabolites of black, red, and non-pigmented (white) rice cultivars (**A**). Detailed correlation matrix of each compound cluster (**B**). Each square indicates a Pearson’s correlation coefficient for a pair of compounds. The value for the correlation coefficient is represented by the intensity of the blue or red color, as indicated on the color scale. Hierarchical clusters are presented as a cluster tree. C20-ol, Eicosanol; C21-ol, Heneicosanol; C22-ol, Docosanol; C24-ol, Tetracosanol; C26-ol, Hexacosanol; C27-ol, Heptacosanol; C28-ol, Octacosanol; C30-ol, Triacontanol; C16:0, Palmitic acid; C18:0, Stearic acid; C18:1, Oleic acid; C18:2, Linoleic acid; C18:3, alpha-Linolenic acid; C20:0, Arachidic acid.

Pearson’s correlation analysis and HCA were performed to understand the relationships and metabolic network formed by the 110 identified metabolites (Figure 3 and Table S8). HCA revealed four metabolite clusters with strong correlations between metabolites and that were involved in closely related metabolic pathways (Figure 3A). Most amino acids, *p*-hydroxybenzoic acid, catechin, and epicatechin were placed together in cluster 1 (Figure 3B). Cluster 2 contained most organic acids, sugars, and fatty acids. Cluster 3 consisted of terpenoids, policosanols, phenolic acids, and anthocyanins. Finally, cluster 4 contained most volatiles, some tocopherols, and sugars (raffinose and mannitol). These four clusters revealed grouping of metabolites with closely related biosynthesis pathways. Furthermore, the metabolite concentrations in each cluster were positively correlated. Most metabolites in clusters 1 and 2 were primary metabolites, whereas those in clusters 3 and 4 were secondary metabolites. In addition, the levels of nitrogen compounds, such as amino acids, in clusters 1 and 2 were positively correlated; however, they were negatively correlated with the content of most carbon metabolites in the correlation matrix. Cellular carbon metabolism and nitrogen metabolism in plants are closely coordinated for optimal growth [24,25]. The above analyses demonstrated the complex interactions between nitrogen and carbon metabolism of primary and secondary metabolites, connected via biochemical networks of metabolic pathways. Most amino acids, catechin, and epicatechin levels in cluster 1 were positively correlated with each other; however, they were negatively correlated with the levels of terpenoids and anthocyanins in clusters 3 and 4, such as tocopherols, carotenoids, phytosterols, peonidin-3-glucoside, and cyanidin-3-glucoside. In addition, the levels of terpenoids and anthocyanins in clusters 3 and 4 were positively correlated. These observations were in agreement with the findings of a previous study reporting that carotenoids and flavonoids are positively correlated [3]. However, in the present study, correlations of the levels of not only carotenoids, but also of terpenoids, such as monoterpenes, phytosterols, and tocopherols, with the levels of flavonoids were determined. The metabolite with the highest correlation coefficient for anthocyanins was β-carotene (peonidin-3-*O*-glucoside, *r* = 0.7162, *p* < 0.0001; cyanidin-3-*O*-glucoside, *r* = 0.7481, *p* < 0.0001) (Table S8). In addition, monoterpenes, such as d-limonene, *p*-cymene, *α*-pinene, linalool, and γ-terpinene, showed positive correlation coefficient values. Among monoterpenes, d-limonene (peonidin-3-*O*-glucoside, *r* = 0.5655, *p* < 0.0001; cyanidin-3-*O*-glucoside, 0.6052, *p* < 0.0001) and *p*-cymene (peonidin-3-*O*-glucoside, *r* = 0.4633, *p* = 0.0005; cyanidin-3-*O*-glucoside, *r* = 0.4857, *p* = 0.0005) showed high correlation coefficient values and were closely clustered with anthocyanins. The highest correlation coefficient between triterpenes and flavonoids was for stigmasterol (peonidin-3-*O*-glucoside, *r* = 0.4343, *p* = 0.002; cyanidin-3-*O*-glucoside, *r* = 0.4412, *p* = 0.0017) and campesterol (peonidin-3-*O*-glucoside, *r* = 0.4226, *p* = 0.0028; cyanidin-3-*O*-glucoside, *r* = 0.4530, *p* = 0.0012). Furthermore, the most significant positive correlation between tetraterpenes and flavonoids was for lutein (peonidin-3-*O*-glucoside, *r* = 0.4245, *p* = 0.0026; cyanidin-3-*O*-glucoside, *r* = 0.4409, *p* = 0.0017) and α-tocopherol (peonidin-3-*O*-glucoside, *r* = 0.3198, *p* = 0.0267; cyanidin-3-*O*-glucoside, *r* = 0.3408, *p* = 0.0178), except for β-carotene (the highest correlation value). According to these correlations between terpenoids and anthocyanins, the BR cultivars showed abundance of terpenoids in proportion to the concentrations of anthocyanins (Tables S9 and S10). The BR cultivars in order of pigment (anthocyanins) concentration were as follows: SW 505 (cyanidin-3-*O*-glucoside, 742.65 ± 15.69 μg/g; peonidin-3-*O*-glucoside, 32.65 ± 0.73 μg/g), HJJ (cyanidin-3-*O*-glucoside, 493.23 ± 30.50 μg/g; peonidin-3-*O*-glucoside, 20.73 ± 0.53 μg/g), and JSHC (cyanidin-3-*O*-glucoside, 267.27 ± 13.27 μg/g; peonidin-3-*O*-glucoside, 14.06 ± 1.46 μg/g) (Table S10). These three BR cultivars showed 2–5-times higher concentrations of monoterpenes as well as of triterpenes and tetraterpenes compared with the respective concentrations in other rice cultivars. On the contrary, the concentrations of catechin and epicatechin showed negative correlation coefficients (*r* = −0.5040 to 0.0090) with those of terpenoids (Table S8). In cluster 2, fatty acids concentrations were negatively correlated with the levels of flavonoids, such as catechin, epicatechin, peonidin-3-glucoside, and cyanidin-3-glucoside. Furthermore, in cluster 3, protocatechuic acid (peonidin-3-*O*-glucoside, *r* = 0.6936, *p* < 0.0001; cyanidin-3-*O*-glucoside, *r* = 0.6654, *p* < 0.0001) and vanillic acid (peonidin-3-*O*-glucoside, *r* = 0.6860, *p* < 0.0001; cyanidin-3-*O*-glucoside, *r* = 0.6079, *p* < 0.0001) concentrations were positively correlated with those of anthocyanins. According to a previous study, soluble free protocatechuic acid and vanillic acid might act as precursors or accelerants in the anthocyanin biosynthesis pathway [26]. According to another study, cyanidin-3-glucoside is deglycosylated upon heating, with subsequent formation of protocatechuic acid upon degradation of deglycosylated cyanidin [27]. Furthermore, vanillic acid might be generated from peonidin-3-glucoside via the same mechanism [28]. Consequently, protocatechuic acid, vanillic acid, and anthocyanins are positively correlated. These observations were also consistent with the PLS-DA loading plot and OPLS-DA VIP plot analysis (Figure 1 and Figure 2). Almost all terpenoids, including monoterpenes, triterpenes, and tetraterpenes, were placed together in cluster 3, except for β-tocopherol, γ-tocopherol, γ-tocotrienol, γ-terpinene, and *o*-cymene. Most volatiles were placed together in cluster 4, except for α-pinene, *p*-cymene, d-limonene, ethylbenzene, and 2-acetyl-1-pyrroline, which clustered together in cluster 3. Monoterpenes in cluster 3, such as α-pinene, *p*-cymene, and d-limonene, were strongly correlated.

### 2.3. PathVisio Pathway Analysis

**Figure 4:**
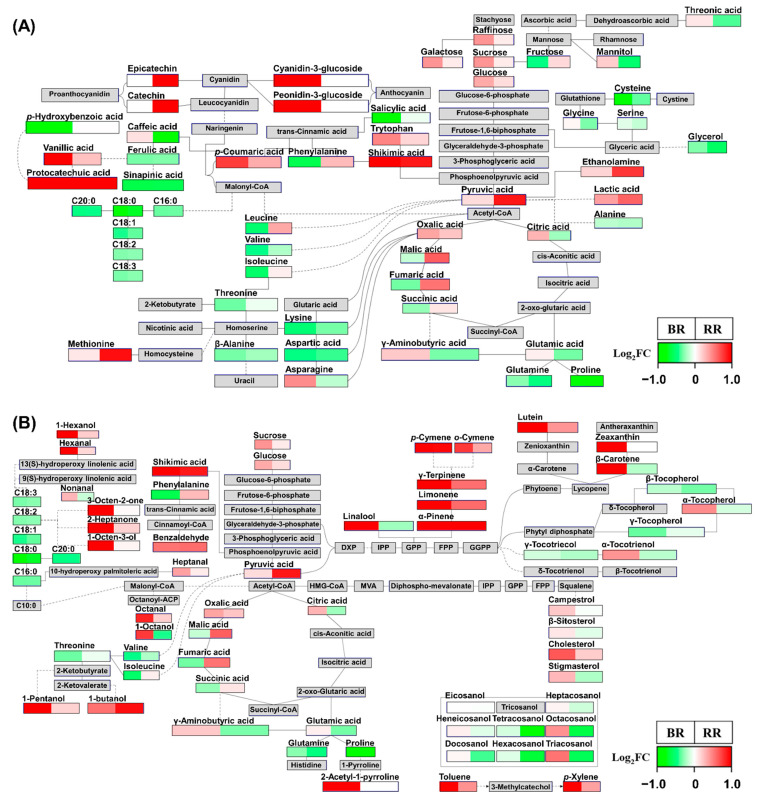
Metabolic pathway diagrams visualized by PathVisio 3. Detailed metabolic pathway diagrams of amino acids, organic acids, sugars, phenylpropanoids, and fatty acids (**A**); terpenoids and volatiles (**B**). The expression data consist of log_2_-transformed fold change (FC) values (log_2_FC). A log_2_FC value range is −1 < log_2_FC < 1. If log_2_FC value is higher than zero (indicated in red), metabolite content is higher in pigmented rice than in non-pigmented rice (WR: white rice). If log_2_FC value is less than zero (indicated in green), metabolite content is lower in pigmented rice than in non-pigmented rice. If log_2_FC value is zero (indicated in white), metabolite content is identical in pigmented and non-pigmented rice. (BR: Black Rice, RR: Red Rice).

To interpret the findings of multivariate statistical analyses described in Section 2.1 and Section 2.2 in the context of metabolic pathways, PathVisio was used to visualize metabolic changes in 110 metabolites in pathway diagrams (18 metabolites are not shown in these diagrams) (Figure 4) [29]. The fold change (FC) was calculated by dividing the average values for pigmented rice by the average values for non-pigmented rice (WR), and then log_2_-transforming (log_2_FC) (Table S11). The log_2_FC data (ranging from −1 to 1) were visualized on pathway diagrams by PathVisio.

Concerning the phenylpropanoid pathway, BR contained more cyanidin-3-glucoside and peonidin-3-glucoside than WR (Figure 4A). On the contrary, RR contained more catechin and epicatechin than WR. These metabolites determine the color of pigmented rice [3,4]. Hence, these observations indicated that the synthesis of color metabolites in pigmented rice proceeds via a different route in the same phenylpropanoid pathway. These findings were in agreement with the HCA and Pearson’s correlation analysis, which indicated that catechin and epicatechin are negatively correlated with anthocyanins (Figure 3). In addition, in PLS-DA and OPLS-DA, catechin and epicatechin were significant contributing metabolites in RR, whereas anthocyanins were important metabolites in BR (Figure 1 and Figure 2). Shikimic acid is the starting point of the phenylpropanoid pathway, which then proceeds to phenylalanine, *trans*-cinnamic acid, and *p*-coumaric acid. These metabolites are important precursors of flavonoids and phenolics, and were more abundant in pigmented rice than in WR, except for phenylalanine (lower levels in BR than in WR) and *trans*-cinnamic acid (not detected). Although BR contained less phenylalanine than WR, *p*-coumaric acid was notably more abundant in BR than in RR and WR. These observations suggested that the high level of *p*-coumaric acid in BR might arise from the biotransformation of phenylalanine, stimulated by upregulation of phenylalanine ammonia-lyase (PAL) activity. PAL is a key enzyme that acts at the first step of the phenylpropanoid pathway, and converts phenylalanine to *trans*-cinnamic acid and ammonia. According to a previous study, PAL activity impacts anthocyanin levels in the flavonoid pathway [30]. In addition, PAL regulates anthocyanin accumulation [31]. A further, genomics, study is needed to address this issue.

Furthermore, fatty acid levels in pigmented rice were lower than those in WR. Malonyl-CoA is the precursor of both, fatty acid and flavonoid biosynthesis. Chalcone synthase (CHS) converts three molecules of malonyl-CoA and one molecule of 4-coumaroyl-CoA into naringenin chalcone [32,33]. Therefore, pigmented rice might consume more malonyl-CoA than WR to synthesize flavonoids for pigment formation, such as black and red. Consequently, pigmented rice might lack malonyl-CoA for fatty acid synthesis. On the contrary, WR does not synthesize flavonoids, and contained relatively more fatty acids than pigmented rice, as explained above. Similarly, wild beans, which contain high levels of flavonoids, have relative lower fatty acid levels than cultivated beans [34]. The above results were consistent with PLS-DA and OPLA-DA (Figure 1 and Figure 2).

As shown in Figure 4B, carotenoids and phytosterols, such as β-carotene, lutein, zeaxanthin, campesterol, β-sitosterol, cholesterol, and stigmasterol, were more abundant in BR than in RR and WR, which was consistent with previous studies reporting the abundance of carotenoids and phytosterols in BR [3,20]. However, in the present study, pigmented rice contained less β-tocopherol, γ-tocopherol, and γ-tocotrienol than WR. In addition, these metabolites contributed to the separation of WR from other rice samples in PLS-DA and OPLS-DA, and they clustered together, separately from other terpenoids. The major tocopherols in rice are α-tocopherol, α-tocotrienol, γ-tocopherol, and γ-tocotrienol [35]. According to a previous study, α-tocopherol levels are highest in BR, [36].

In addition, most volatiles, such as monoterpenes, fatty acid-driven volatiles, and other volatiles, were most abundant in BR. Therefore, BR exhibited higher secondary metabolic activity, such as that involving terpenoid (monoterpene, triterpene, and tetraterpene) and phenylpropanoid (anthocyanin) biosynthesis, than RR and WR. To the best of our knowledge, this is the first report on the differences in terpenoid metabolism in non-pigmented and pigmented rice associated with their color.

**Figure 5:**
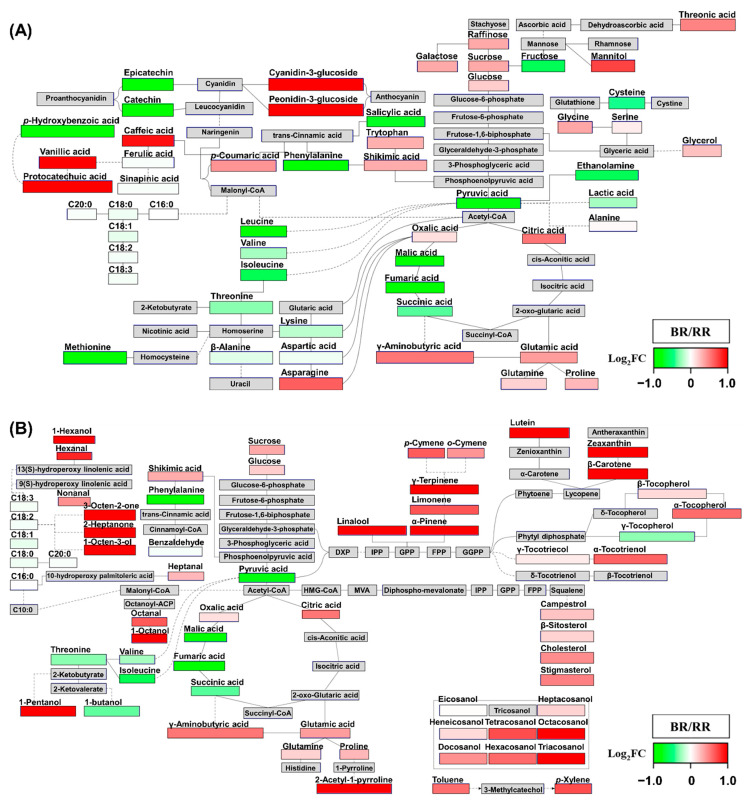
Metabolic pathway diagrams visualized by PathVisio 3. Detailed metabolic pathway diagrams of amino acids, organic acids, sugars, phenylpropanoids, and fatty acids (**A**); terpenoids and volatiles (**B**). The expression data consist of log_2_-transformed fold change (FC) values (log_2_FC). A log_2_FC value range is −1 < log_2_FC < 1. If log_2_FC value is higher than zero (indicated in red), metabolite content is higher in black rice than in red rice. If log_2_FC value is less than zero (indicated in green), metabolite content is higher in red rice than in black rice. If log_2_FC value is zero (indicated in white), metabolite content is identical in black and red rice. (BR: Black Rice, RR: Red Rice).

To elucidate the metabolic differences between pigmented rice samples, FC was calculated by dividing the average data of BR by the average data of RR, and then log_2_-transforming (log_2_FC) (Table S11). The log_2_FC data (ranging from −1 to 1) were visualized on pathway diagrams in PathVisio (Figure 5). Most amino acids and organic acids were more abundant in RR than in BR, except for γ-aminobutyric acid, asparagine, proline, glutamine, tryptophan, glutamic acid, oxalic acid, and citric acid (Figure 5A). Furthermore, most organic acids and amino acids significantly contributed to the separation of RR from BR in the PLS-DA loading plot and OPLS-DA VIP plot (Figure 1 and Figure 2). Among “other” metabolites, γ-aminobutyric acid and asparagine are reportedly highly abundant in BR [37,38], which is consistent with the data presented herein. In addition, the *t*-test *p*-value for these metabolites comparing BR and RR was below 0.05, and these metabolites contributed to the separation of BR from RR in the PLS-DA loading plot and OPLS-DA VIP plot.

Protocatechuic acid was not detected in RR in one study [26]; however, here, we have detected this metabolite in RR (Figure 5A). In HCA, cluster 3 revealed a positive correlation of vanillic acid, protocatechuic acid, and anthocyanins, and these metabolites were more abundant in BR than in RR (Figure 3 and Figure 5A). In addition, these metabolites significantly contributed to the separation of BR from others in PLS-DA and OPLS-DA (Figure 1 and Figure 2). These observations supported the notion that vanillic acid and protocatechuic acid are the major precursors or accelerants of the anthocyanin biosynthesis pathway [26].

Most volatiles, except for 1-butanol, were more abundant in BR than in RR. Specifically, fatty acid-derived volatiles, such as 1-hexanol, 2-heptanone, hexanal, 3-octen-2-one, 1-octen-3-ol, and nonanal, were notably more abundant in BR than in RR (Figure 5B and Table S5). These observations were consistent with previous studies, which showed that fatty acid-derived volatiles are abundant in BR [9,16].

Considering the terpenoid biosynthesis pathway, carotenoid, tocopherol, phytosterol, and monoterpene levels were higher in BR than in RR. Furthermore, policosanols were more abundant in BR than in RR. In multivariate statistical analysis, these metabolites significantly contributed to the separation of BR from other rice samples, and were positively correlated.

To sum up, BR has active secondary metabolism responsible for the black color, such as terpenoid (carotenoid, tocopherol, phytosterol, and monoterpene), anthocyanin, policosanol, and fatty acid-derived volatile metabolism. By contrast, from the secondary metabolic pathways, RR only exhibited active phenylpropanoid metabolism for the synthesis of red color compounds. Therefore, BR contained high amounts of carbon-based secondary metabolites, with relatively lower levels of primary metabolites, such as amino acids and organic acids, than those in other rice samples. The primary metabolites are the building blocks for the synthesis of secondary metabolites. Hence, BR contained only low levels of most of the primary metabolites. In addition, BR contained high levels of sugars, except for fructose, because they are important carbon sources for secondary metabolism starting from glycolysis. Specifically, sucrose and genes associated with sucrose metabolism modulate phenylpropanoid metabolism and induce flavonoid production and accumulation in various plants [39,40].

### 2.4. Quality Assessment of Pigmented Rice

The statistical analysis presented in the preceding sections focused on a comprehensive analysis of metabolic differences among pigmented rice cultivars associated with their color. However, these analyses do not provide information on the nutritional composition of individual rice cultivars. Therefore, we performed quality assessment to identify rice cultivars containing high amounts of bioactive compounds. SW 505 (BR) rice cultivar had the highest anthocyanin content of all samples (cyanidin-3-*O*-glucoside, 742.65 ± 15.69 μg/g; penonidin-3-*O*-glucoside, 32.65 ± 0.73 μg/g) (Table S9). The concentration of catechin and epicatechin was generally the highest in AM (RR) rice cultivars (catechin, 630.85 ± 152.48 μg/g; epicatechin, 29.11 ± 1.40 μg/g). The volatiles were most abundant in most BR cultivars (Table S3). Specifically, the levels of volatiles, such as hexanal (57.56 ± 3.13 × 10^8^ area/g), nonanal (28.46 ± 1.37 × 10^8^ area/g), 1-hexanol (164.37 ± 5.47 × 10^8^ area/g), 2-heptanone (14.30 ± 0.80 × 10^8^ area/g), and styrene (41.81 ± 5.27 × 10^8^ area/g) were the highest in JSHC (Table S12). In addition, 2-acetyl-1-pyrroline, responsible for the major flavor preferred by the customer, was only detected in HJJ and HH (Table S12).

## 3. Materials and Methods

### 3.1. Samples and Chemicals

The 16 cultivars of rice (*Oryza sativa* L.) analyzed in the present study were categorized as non-pigmented (white), black, or red, in accordance with their pericarp color (Figure S3), as follows: WR: Purple check (PC), Dongjin (DJ), and Heugdaegu (HDG); BR: Heughyang (HH), Heugjinju (HJJ), Heugnam (HN), Josengheugchal (JSHC), Maligate Pirurutong (MP), Suwon 493 (SW 493), and Suwon 505 (SW 505); RR: Aengmi (AM), Goryeong 8 (GR8), Hongjinju (HoJJ), Hanyangjo (HYJ), Siga-Chata (SC), and Jagwangdo (JGD). The rice samples were obtained from the Agricultural Genetic Resources Center at the National Academy of Agricultural Science (Suwon, Korea). The seeds were harvested in 2016 and the rice samples were manually hulled. The samples were pulverized on the same day using a mortar and a pestle. The rice powder was stored in a refrigerator at −20 °C prior to analysis. Pyridine, *N*-methyl-*N*-(trimethylsilyl) trifluoroacetamide (MSTFA), methoxyamine hydrochloride, ribitol, 5α-cholestane, and fatty acid methyl ester (FAME) mixture (C_8_–C_24_) were obtained from Sigma-Aldrich Corp. (St. Louis, MO, USA). Peonidin-3-*O*-glucoside and cyanidin-3-*O*-glucoside were purchased from Extrasynthese (Genay, France). All chemicals and reagents used in the study were HPLC grade.

### 3.2. Extraction and Analysis of Hydrophilic Compounds

Hydrophilic compounds (amino acids, organic acids, sugars, phenolic acids, and sugar alcohols) were extracted as previously described [29]. Briefly, 100 mg of ground rice samples were placed in 2 mL tubes with 1 mL of methanol:chloroform:water (2.5:1:1, *v/v*/*v*) solution. Then, 60 μL of ribitol (200 μg/mL in methanol) was added as an internal standard (IS). After vortexing, the samples were incubated at 37 °C for 30 min, with shaking at 1200 rpm. Next, the mixtures were centrifuged at 16,000× *g* for 3 min at 4 °C. The supernatant (800 μL) was transferred to a new 2 mL tube and mixed with 400 μL of deionized water. The samples were centrifuged again at 16,000× *g* for 3 min at 4 °C, and 900 μL of the supernatant were transferred to a new tube. The solvent was completely evaporated in a centrifugal concentrator (CC-105, TOMY, Tokyo, Japan) for 3 h and freeze-dried at −80 °C for at least 16 h. For derivatization, the samples were treated with 80 μL of methoxyamine hydrochloride in pyridine (20 mg/mL) and incubated at 30 °C for 90 min in a thermomixer (model 5355, Eppendorf AG, Hamburg, Germany), with shaking at 1200 rpm. Next, 80 μL of MSTFA was added, and the samples incubated at 37 °C for 30 min, with shaking at 1200 rpm. The derivatized samples (80 μL) were transferred to glass inserts in GC auto sampler vials. The hydrophilic compounds were analyzed using Agilent 7890A GC (Agilent, Santa Clara, CA, USA) coupled to a Pegasus 4D TOF-MS (LECO, St. Joseph, MI, USA). Helium gas was passed at a rate of 1.20 mL/min. Thereafter, 1 μL of the extracted sample was injected in a 1:25 ratio split mode, and the inlet temperature was set at 250 °C. Rtx-5 MS (0.25 mm × 0.25 µm × 30 m; Restek, Bellefonte, PA, USA) and Rxi-17sil MS (0.15 mm × 0.15 µm × 1.2 m; Restek, Bellefonte, PA, USA) were used as the first and second column, respectively, for GC×GC-TOF-MS. The conditions for each column were set individually. The oven temperature was programmed as follows: the oven temperature for the first column was maintained at 80 °C for 0.5 min, followed by ramping to 330 °C at 5 °C/min, and then holding at this temperature for 5 min. The oven temperature for the second column was set with 5 °C offset from the first column temperature. The modulator temperature program was 15 °C offset above the second column temperature. The modulation period was set to 4 s, with 0.6 s hot and 1.4 s cool pulse durations. The ion source and transfer line temperatures were set at 230 °C and 260 °C, respectively. The data were acquired over an *m/z* mass range of 45–650, and the detector voltage was set to 1700 V. Qualitative analysis of peaks was performed using the Chroma TOF software (version 4.5, LECO, St. Joseph, MI, USA), and peaks were identified based on the mass spectral data by comparing with in-house libraries, NIST, and Wiley9 (Figure S4 and Table S13). The quantitative estimation was based on peak area ratios relative to the IS peak area (Table S2).

### 3.3. Extraction and Analysis of Lipophilic Compounds

#### 3.3.1. Extraction and Analysis of Terpenoids and Policosanols Using GC-qMS

Lipophilic compounds, such as policosanols, tocopherols, and phytosterols, were extracted following the method of Kim et al. [41]. Briefly, 100 mg of pigmented rice powder were transferred to 15 mL tubes. For the extraction, ascorbic acid (3 mL; 0.1%, *w/v*) in ethanol was added, with 0.05 mL of 5α-cholestane (10 μg/mL in hexane) as an IS. The samples were vortexed for 20 s, and incubated in a water bath at 85 °C for 5 min. Saponification with potassium hydroxide (120 μL; 80%, *w/v*) was conducted in a water bath at 85 °C for 10 min. The mixture was then immediately placed on ice for 5 min. It was then treated with hexane and deionized water (1.5 mL each), and centrifuged at 4 °C and 1200× *g* for 5 min. The upper layer was pipetted into a new tube and re-extracted with hexane (1.5 mL). The hexane fraction (approximately 3 mL) was dried under nitrogen gas, and then concentrated in a centrifugal concentrator (CC-105, TOMY, Tokyo, Japan). For derivatization, MSTFA (30 μL) and pyridine (30 μL) were added, and the samples incubated at 60 °C for 30 min, with shaking at 1200 rpm. The obtained lipophilic compounds were analyzed using a GC-MS QP2010 Ultra system equipped with an AOC-20i auto sampler (Shimadzu, Kyoto, Japan). For the analysis, 1 μL of the extracted sample was injected into a Rtx-5MS column (30 m × 0.25 mm × 0.25 μm; Restek, Bellefonte, PA, USA) at a 1:10 ratio split mode. The carrier gas was helium, flowing at a constant rate of 1.00 mL/min. The front inlet temperature was 290 °C. The initial oven temperature of 150 °C was held for 2 min, then ramped to 320 °C at 15 °C/min, and maintained for 10 min. The ion source and interface temperatures were 230 °C and 250 °C, respectively. The mass spectra range for scanning was 85 to 600 *m/z*, and the ions were detected in selected-ion monitoring (SIM) mode for peak analysis. Chromatographic data were processed using the Lab solutions GC-MS solution software (version 4.11, Shimadzu, Kyoto, Japan). For qualitative and quantitative analysis of the lipophilic compounds, calibration curves were prepared using lipophilic standards (Tables S4 and S10).

#### 3.3.2. Extraction and Analysis of Fatty Acids Using GC-FID

To determine the fatty acid composition, the extraction and analysis were performed as previously described [29]. Briefly, 10 mg of pigmented rice powder were mixed with 2.5 mL of a chloroform:methanol solution (2:1, *v/v*) and 0.1 mL of pentadecanoic acid (1 mg/mL in chloroform; used as an IS). The samples were vortexed and sonicated for 20 min. Then, 2.5 mL of sodium chloride solution (0.58%, *w/v*) was added, and the mixtures were centrifuged at 4 °C, 13,000× *g* for 5 min. The bottom layer was collected into new tubes. The transferred samples were then evaporated in a centrifugal concentrator for 30 min. Thereafter, 0.18 mL of methanol, 0.1 mL of toluene, and 0.02 mL of 5 M sodium hydroxide in water were added, and the samples heated at 85 °C for 5 min, with shaking at 300 rpm. For the saponification and methylation, 0.3 mL of 14% (*v/v*) boron trifluoride (BF3) was added, and the samples incubated at 85 °C for 5 min, with shaking at 300 rpm. After cooling at 25 °C, 400 μL of distilled water and 800 μL of pentane were added, and the samples centrifuged at 750× *g* for 15 min at 4 °C. The upper pentane layer was transferred into 2 mL tubes and concentrated in a rotary evaporator (CC-105, TOMY, Tokyo, Japan). Prior to the analysis, the concentrated samples were diluted in 100 μL of hexane, and then filtered through a 0.5 μm syringe filter. The samples were analyzed using Agilent 7890B GC-FID (Agilent, Santa Clara, CA, USA) equipped with Agilent G4513A auto sampler (Agilent, Santa Clara, CA, USA). For the analysis, 1 μL of the extracted sample was injected into the column at a 1:10 ratio split mode. The front inlet and detector temperatures were 250 °C. The GC was equipped with a DB-WAX column (30 m × 0.25 mm × 0.25 μm, Agilent, Santa Clara, CA, USA), and nitrogen was used as a carrier gas, at a flow rate of 1.00 mL/min. The column oven temperature was maintained at 130 °C for 3 min, and was then increased at a rate of 20 °C/min until it reached 230 °C. The temperature was then increased to 250 °C at a rate of 3 °C/min, and finally maintained at 250 °C for 5 min. The oven post-run time was 5 min. The peak data were acquired using the ChemStation software (Agilent, Santa Clara, CA, USA) (Figure S5). Qualitative and quantitative analyses of FAME were done using standards and FAME mixture (C_8_–C_24_) (Tables S10 and S14).

#### 3.3.3. Extraction and Analysis of Carotenoids Using HPLC

The extraction method was as previously described by Kim et al. [29]. Briefly, carotenoids were released from the pigmented rice powder (300 mg) by adding 3 mL of ethanol containing 0.1% ascorbic acid (*w/v*), and the mixture was vortexed for 20 s. The samples were placed in a water bath at 85 °C for 5 min. The carotenoid extract was saponified with potassium hydroxide (120 μL; 80%, *w/v*) at 85 °C for 10 min in a water bath. After saponification, the samples were immediately placed on ice. Then, 100 μL of β-apo-8′-carotinal (25 μg/mL in ethanol) was added as an IS. Deionized water and hexane (1.5 mL each) were added to the samples, which were then vortexed for 20 s and centrifuged at 4 °C and 1200× *g* for 5 min. The supernatant was transferred into a new tube, and the extraction was repeated with hexane (1.5 mL). The hexane fraction was evaporated under nitrogen, and then dissolved in 250 μL of 50:50 dichloromethane/methanol (*v/v*) before HPLC analysis. The carotenoids were separated on a C30 YMC column (250 × 4.6 mm, 3 μm; YMC Co., Kyoto, Japan) by HPLC (Agilent 1100 HPLC instrument, Santa Clara, CA, USA) equipped with a diode-array detector. The detector wavelength was set to 450 nm and the column temperature was 40 °C. Solvent A consisted of methanol/water (92:8, *v/v*) with 10 mM ammonium acetate. Solvent B consisted of 100% methyl *tert*-butyl ether. A binary gradient elution system of Solvent A-Solvent B was set, as follows: 0 min, 90% A/10% B; 20 min, 83% A/17% B; 29 min, 75% A/25% B; 35 min, 30% A/70% B; 40 min, 30% A/70% B; 42 min, 25% A/75% B; 45 min, 90% A/10% B; 55 min, 90% A/10% B. For quantification, a calibration curve was prepared using standard compounds, and the quantities were calculated as the ratio of the peak area of the standard compound to the peak area of the IS (Figure S6 and Table S10).

### 3.4. Extraction and Analysis of Anthocyanins

Anthocyanins were extracted according to the method of Kim et al. [3], with a slight modification. Briefly, 50 mg of pigmented rice powder was placed in 2 mL tubes, and 0.8 mL of 85% methanol acidified with 1.0 N HCl solution was added to assist the extraction. The samples were then sonicated for 1 min and centrifuged to separate the layers (4 °C, 10,000× *g*, 5 min). The supernatant (800 μL) was transferred to a new 2 mL tube and stored below −20 °C. The extraction process was repeated, and the new supernatant (800 μL) was collected and combined with the supernatant stored in the freezer. The extracts were then incubated at 38 °C for 30 min, with a mixing frequency of 500 rpm, using a Thermomixer Compact (Eppendorf AG, Hamburg, Germany). The crude extract was passed through a 0.22 μm Teflon PTFE syringe filter before HPLC analysis. Anthocyanins were separated on a C18 column (250 mm, 4.6 mm, 5 μm, Inertsil ODS-3, GL Sciences, Tokyo, Japan) by using Waters Alliance e2695 HPLC (Waters Corporation, Milford, MA, USA) equipped with a 2998 photodiode array detector. Elution was performed using a binary gradient of 0.1% formic acid in water (mobile phase A) and 0.1% formic acid in acetonitrile (mobile phase B) according to the following program: 0 min, 95% A/5% B; 40 min, 50% A/50% B; 42 min, 0% A/100% B; 52 min, 0% A/100% B; 54 min, 95% A/5% B; and 64 min, 95% A/5% B. The flow rate was 1.0 mL/min, and the column temperature was 40 °C. The UV-vis detector wavelength was set to 520 nm. The qualitative analysis of cyanidin-3-*O*-glucoside and peonidin-3-*O*-glucoside was performed using the retention time of the standard material (Figure S7). Quantification was done using a standard curve drawn as the peak area to the concentration of each standard (1.25–62.5 μg) (Table S9).

### 3.5. Extraction and Analysis of (+)-Catechin and (−)-Epicatechin

Catechin and epicatechin were extracted and analyzed as previously reported [42], with slight modification. Powdered rice samples (0.01 g) were extracted with 200 μL of 80% ethanol containing 1.2 M HCl. After vortexing, the samples were incubated at 30 °C for 2 h, with shaking at 1200 rpm, and centrifuged at 16,000× *g* for 10 min at 4 °C to separate the layers. The extract was filtered through a 0.5 μm syringe filter, transferred into a 2 mL vial, and analyzed by LC-MS. The LC-MS analysis was performed using an Agilent 1260 Infinity HPLC System (degasser, quaternary pump, auto sampler, an Agilent 6120 single quadrupole MS with electrospray ionization; Open LAB CDS ChemStation Edition Rev. C.01.07 software (Agilent Technologies, Santa Clara, CA, USA). Elution was performed using a binary gradient of 0.1% formic acid in water (mobile phase A) and 0.1% formic acid in acetonitrile (mobile phase B), according to the following program: 0 min, 92% A/8% B; 7 min, 90% A/10% B; 15 min, 85% A/15% B; 20 min, 75% A/25% B; 40 min, 70% A/30% B; 45 min, 0% A/100%; and 55 min, 0% A/100% B. The separation was achieved using a Develosil ODS-UG-5 column (2.0 × 250 mm, Nomura Chemical, Seto, Japan) at a flow rate of 0.4 mL/min. A 5 μL portion of each extract was injected for analysis. The analysis conditions of the electrospray ionization MS were as follows: negative ionization mode; dry gas (N_2_), 12 L/min; nebulizer pressure, 35 psig; drying gas temperature, 350 °C; capillary voltage, 3000 V; fragmentor voltage, 120 V; SIM mode, [M–H]^−^ *m*/*z* 289 (catechin, epicatechin). Catechin and epicatechin in pigmented rice grains were determined based on the retention times of the standards and fragmentation pattern (Figure S8). The quantification was performed using the MS peak area of each standard; seven-point analytical curves were prepared (0.078125–5 μg/mL), and a high linearity of *r*^2^ > 0.999 was steadily obtained (Table S3).

### 3.6. Headspace-SPME (HS-SPME) and Analysis of Volatile Compounds

Volatile organic compounds were analyzed using HS-SPME with a divinylbenzene/carboxen/polydimethylsiloxane (DVB/CAR/PDMS) StableFlex fiber (50/30 µm thick, 2 cm long; model 57348-U; Supelco Inc., Bellefonte, PA, USA). The volatile compounds were extracted as reported previously [29], with some modifications. Briefly, a sample of ground rice (0.5 g) was placed in a 10 mL crimp-type HS vial and analyzed using a GC-TOF-MS (Pegasus BT Flux, LECO, St. Joseph, MI, USA). Before analysis, the SPME fiber was conditioned at 270 °C for 30 min. Then, the vial containing the rice sample was heated in an oven at 80 °C for 5 min, with a desorption time of 3 min. The injection port, ion source, and transfer line temperatures were set to 250 °C, 230 °C, and 250 °C, respectively. Helium was used as the carrier gas, and the flow rate was 1.00 mL/min. Individual samples were automatically injected into an Rtx-5MS column (30 m × 0.25 mm × 0.25 μm; Restek, Bellefonte, PA, USA) at a splitless mode. The GC oven temperature program was set as follows: the initial oven temperature was set to 40 °C, held for 1 min, then increased to 250 °C at 8 °C/min, and maintained for 10 min. The data were acquired over an *m/z* mass range of 35–400, and the acquisition rate was 20 spectra/s. The data were analyzed using the Chroma TOF software (version 5.50, LECO, St. Joseph, MI, USA), and the peaks were identified based on the MS data by comparing with in-house libraries, NIST, and Wiley9 (Figure S9 and Tables S5 and S12).

### 3.7. Statistical Analysis

All analyses were performed at least in triplicate. PCA, PLS-DA, and OPLS-DA (SIMCA-P version 13.0; Umetrics, Umeå, Sweden) were used to analyze metabolite profile data obtained from GC×GC-TOF-MS, GC-qMS, GC-FID, HPLC, and LC-MS, for an overview of the relationship of the 16 pigmented rice cultivars and their metabolites. All data were transformed with unit variance scaling, before multivariate analysis. PCA, PLS-DA, and OPLS-DA were based on the calculated eigenvectors and eigenvalues (Tables S1, S6, S7 and S15). PCA, PLS-DA, and OPLS-DA score plots were used to visualize the grouping of samples, and loading plots explained the separation of groups in the score plots. Pearson’s correlation analysis was performed by using the SAS 9.4 software package (SAS Institute, Cary, NC, USA), and HCA of the correlation coefficients was performed using the Multi-Experiment Viewer software version 4.9.0 (http://www.mev.tm4.org/ (accessed on 8 June 2021)) (Table S7). PathVisio (version 3.3.0) was used to visualize metabolic pathways reflecting the experimental data. The biological pathways were drawn based on AtMetExpress overview pathway of *Arabidopsis thaliana* in WikiPathways (https://www.wikipathways.org/ (accessed on 8 June 2021)). All metabolite profiling data were calculated as FC and log_2_-transformed (log_2_FC) (Table S11).

## 4. Conclusions

In the present study, we conducted comprehensive metabolite profiling of 16 rice cultivars based on GC×GC-TOF-MS, HS-SPME-GC-TOF-MS, GC-qMS, GC-FID, HPLC-MS, and HPLC-UV analyses. We identified 110 metabolites, including amino acids, organic acids, sugars, sugar alcohols, phenolic acids, flavonoids, anthocyanins, carotenoids, phytosterols, policosanols, tocopherols, fatty acids, and volatiles. BR contained high levels of metabolites from the terpenoid and phenylpropanoid biosynthesis pathways, whereas RR only contained high levels of metabolites from the phenylpropanoid biosynthesis pathway. Furthermore, WR contained low levels of secondary metabolites. Metabolome profiling was achieved by multivariate statistical analysis and PathVisio (metabolic pathway analysis), to determine the relationship between the rice cultivars and their metabolites. The multivariate and pathway analyses revealed correlations and metabolic differences associated with common and closely linked biosynthesis pathways. For the first time, the relationships and metabolic differences in terpenoid (monoterpenes, triterpenes, and tetraterpenes) content were demonstrated between non-pigmented and pigmented rice. Furthermore, the complex interactions between nitrogen and carbon metabolism of primary and secondary metabolites, via metabolic networks, in rice were demonstrated. These findings provide major new insights for understanding the metabolic networks in WR, BR, and RR, and should support future breeding programs for new rice cultivars.

## Data Availability

The data presented in this study are contained within the article or supplementary material.

## Supplementary Materials

The following are available online at https://www.mdpi.com/article/10.3390/metabo11060367/s1, Figure S1: Principal component analysis (PCA) score plots (A) and loading plots (B) derived from 110 metabolites in 16 rice cultivars, Figure S2: Permutation test by OPLS-DA of pigmented rice and non-pigmented rice (A), and black rice and red rice (B), Figure S3: Phenotypes of 16 rice cultivars used in this study, Figure S4: GCxGC-TOF-MS analytical ion chromatogram (AIC) of hydrophilic compounds extracted from Dongjin (DJ), Figure S5: GC-FID chromatogram of fatty acid compounds from Suwon 505 (SW 505), Figure S6: HPLC chromatogram of carotenoid compounds from Heugnam (HN), Figure S7: HPLC chromatogram of anthocyanin compounds from Heugjinju (HJJ), Figure S8: HPLC chromatogram of catechin and epicatechin from Aengmi (AM), Figure S9: HS-SPME GC-TOF-MS analytical ion chromatogram of volatile compounds from Heugjinju (HJJ), Table S1: PLS-DA loading and VIP of variables, Table S2: Composition and content (ratio/g) of hydrophilic compounds in 16 pigmented rice cultivars, Table S3: Composition and content (µg/g) of catechin and epicatechin in 16 pigmented rice cultivars, Table S4: Relative retention times (RRT) and mass spectral data of lipophilic compounds as trimethylsilyl derivatives, Table S5: Composition and content (original peak areas × 10^8^) of volatiles compounds in 16 pigmented rice cultivars, Table S6: VIP values of OPLS-DA (pigmented rice and non-pigmented rice), Table S7: VIP values of OPLS-DA (black rice and red rice), Table S8: Results of Pearson’s correlation analysis, Table S9: Composition and content (µg/g) of anthocyanins in 16 pigmented rice cultivars, Table S10: Composition and content (µg/g) of lipophilic compounds, fatty acids, and carotenoids in 16 pigmented rice cultivars, Table S11: Pubchem CID and log_2_-transformed fold change (FC) (Log_2_FC) of metabolites, Table S12: Retention times (RT) and mass spectral data for volatile compounds, Table S13: Relative retention times (RRT) and mass spectral data for hydrophilic compounds as trimethylsilyl derivatives, Table S14: Relative retention times (RRT) and concentrations of fatty acid methyl ester (FAME) mixture and fatty acids, Table S15: Loadings of the variables in the first two principal components.
