# Supplementary material for: Metabolite Profiling Reveals Distinct Modulation of Complex Metabolic Networks in Non-Pigmented, Black, and Red Rice (Oryza sativa L.) Cultivars
Source: Metabolites. 2021 Jun 9;11(6):367. doi: 10.3390/metabo11060367 (PMC8230048; doi:10.3390/metabo11060367)
Supplement: Supplementary file 1 [file metabolites-11-00367-s001.zip › PR_Metabolites_proof_supplementary figure_ver1.pptx]

## Slide 1
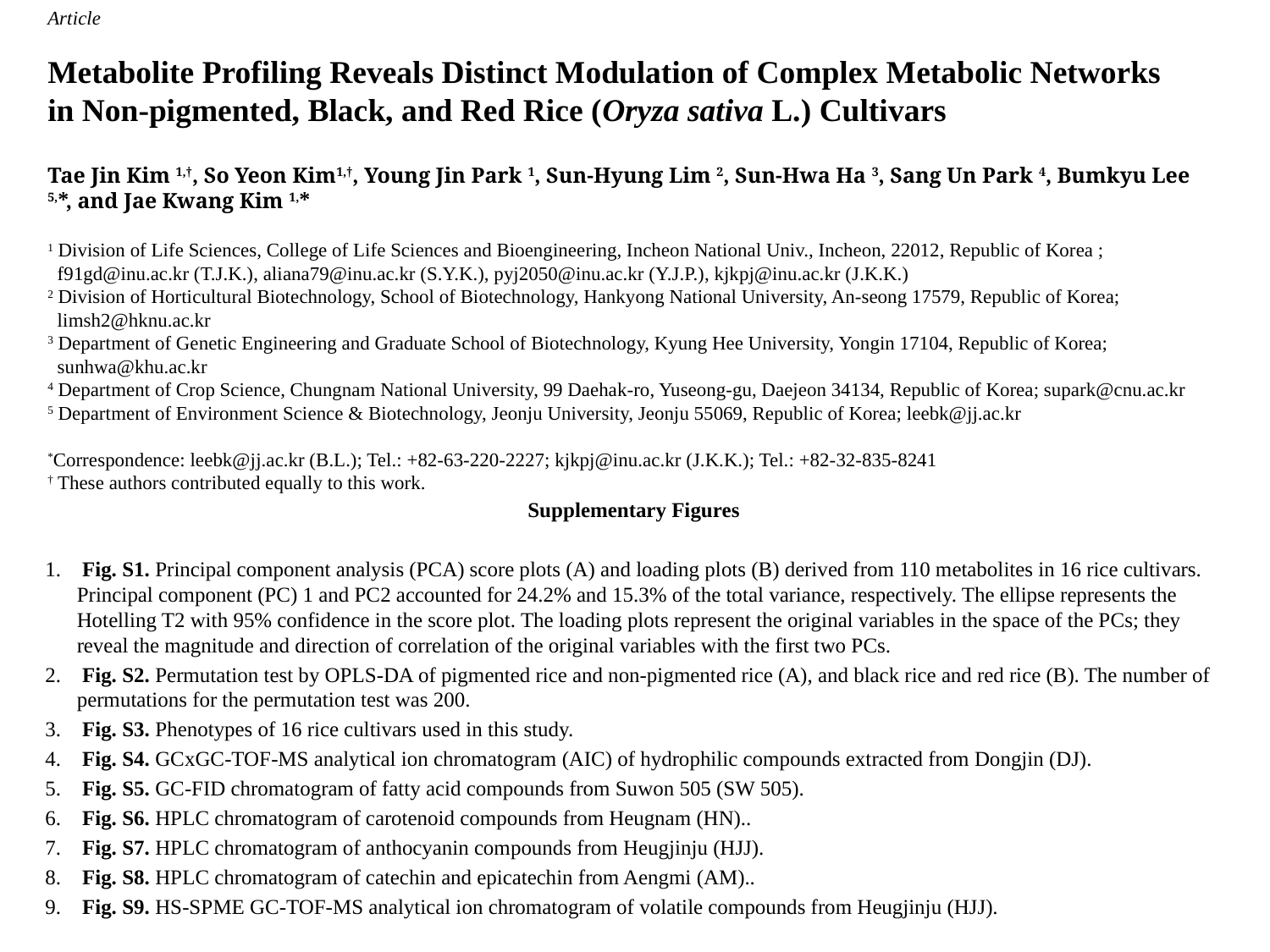

# Article Metabolite Profiling Reveals Distinct Modulation of Complex Metabolic Networks in Non-pigmented, Black, and Red Rice (Oryza sativa L.) Cultivars   Tae Jin Kim 1,†, So Yeon Kim1,†, Young Jin Park 1, Sun-Hyung Lim 2, Sun-Hwa Ha 3, Sang Un Park 4, Bumkyu Lee 5,*, and Jae Kwang Kim 1,*  1 Division of Life Sciences, College of Life Sciences and Bioengineering, Incheon National Univ., Incheon, 22012, Republic of Korea ; f91gd@inu.ac.kr (T.J.K.), aliana79@inu.ac.kr (S.Y.K.), pyj2050@inu.ac.kr (Y.J.P.), kjkpj@inu.ac.kr (J.K.K.) 2 Division of Horticultural Biotechnology, School of Biotechnology, Hankyong National University, An-seong 17579, Republic of Korea; limsh2@hknu.ac.kr 3 Department of Genetic Engineering and Graduate School of Biotechnology, Kyung Hee University, Yongin 17104, Republic of Korea; sunhwa@khu.ac.kr 4 Department of Crop Science, Chungnam National University, 99 Daehak-ro, Yuseong-gu, Daejeon 34134, Republic of Korea; supark@cnu.ac.kr 5 Department of Environment Science & Biotechnology, Jeonju University, Jeonju 55069, Republic of Korea; leebk@jj.ac.kr *Correspondence: leebk@jj.ac.kr (B.L.); Tel.: +82-63-220-2227; kjkpj@inu.ac.kr (J.K.K.); Tel.: +82-32-835-8241 † These authors contributed equally to this work.
Supplementary Figures
 Fig. S1. Principal component analysis (PCA) score plots (A) and loading plots (B) derived from 110 metabolites in 16 rice cultivars. Principal component (PC) 1 and PC2 accounted for 24.2% and 15.3% of the total variance, respectively. The ellipse represents the Hotelling T2 with 95% confidence in the score plot. The loading plots represent the original variables in the space of the PCs; they reveal the magnitude and direction of correlation of the original variables with the first two PCs.
 Fig. S2. Permutation test by OPLS-DA of pigmented rice and non-pigmented rice (A), and black rice and red rice (B). The number of permutations for the permutation test was 200.
 Fig. S3. Phenotypes of 16 rice cultivars used in this study.
 Fig. S4. GCxGC-TOF-MS analytical ion chromatogram (AIC) of hydrophilic compounds extracted from Dongjin (DJ).
 Fig. S5. GC-FID chromatogram of fatty acid compounds from Suwon 505 (SW 505).
 Fig. S6. HPLC chromatogram of carotenoid compounds from Heugnam (HN)..
 Fig. S7. HPLC chromatogram of anthocyanin compounds from Heugjinju (HJJ).
 Fig. S8. HPLC chromatogram of catechin and epicatechin from Aengmi (AM)..
 Fig. S9. HS-SPME GC-TOF-MS analytical ion chromatogram of volatile compounds from Heugjinju (HJJ).

## Slide 2
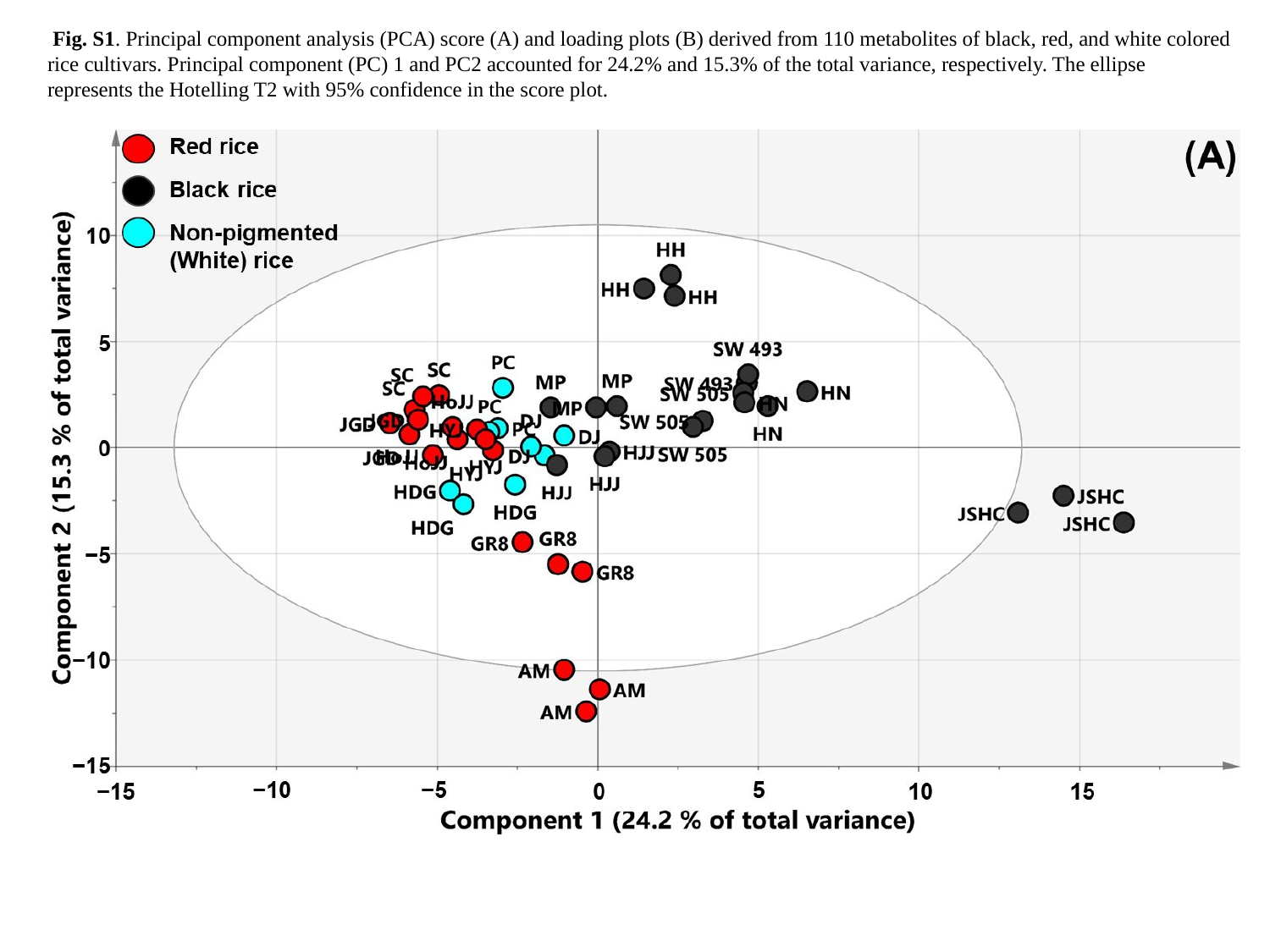

Fig. S1. Principal component analysis (PCA) score (A) and loading plots (B) derived from 110 metabolites of black, red, and white colored rice cultivars. Principal component (PC) 1 and PC2 accounted for 24.2% and 15.3% of the total variance, respectively. The ellipse represents the Hotelling T2 with 95% confidence in the score plot.

## Slide 3
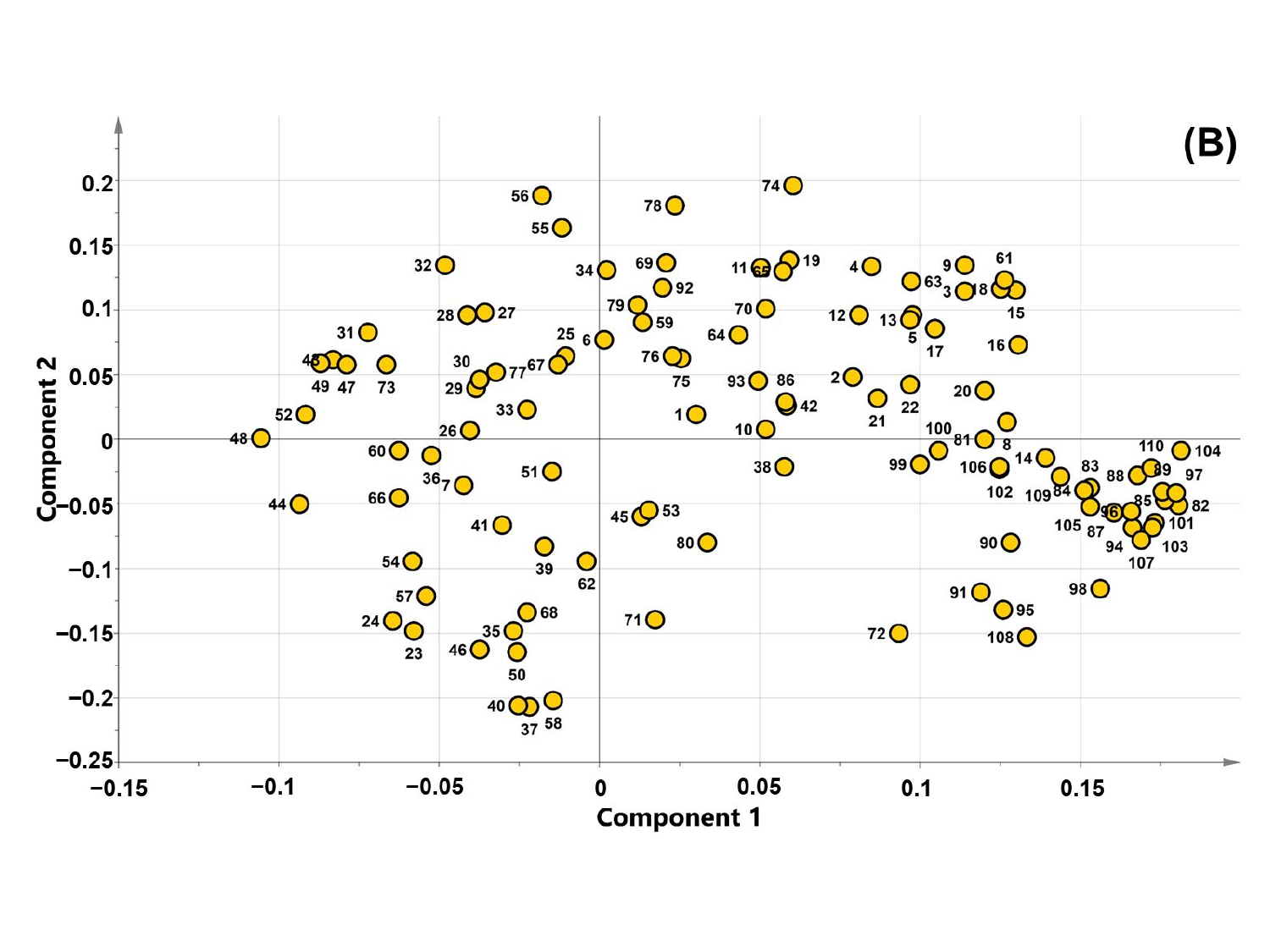

## Slide 4
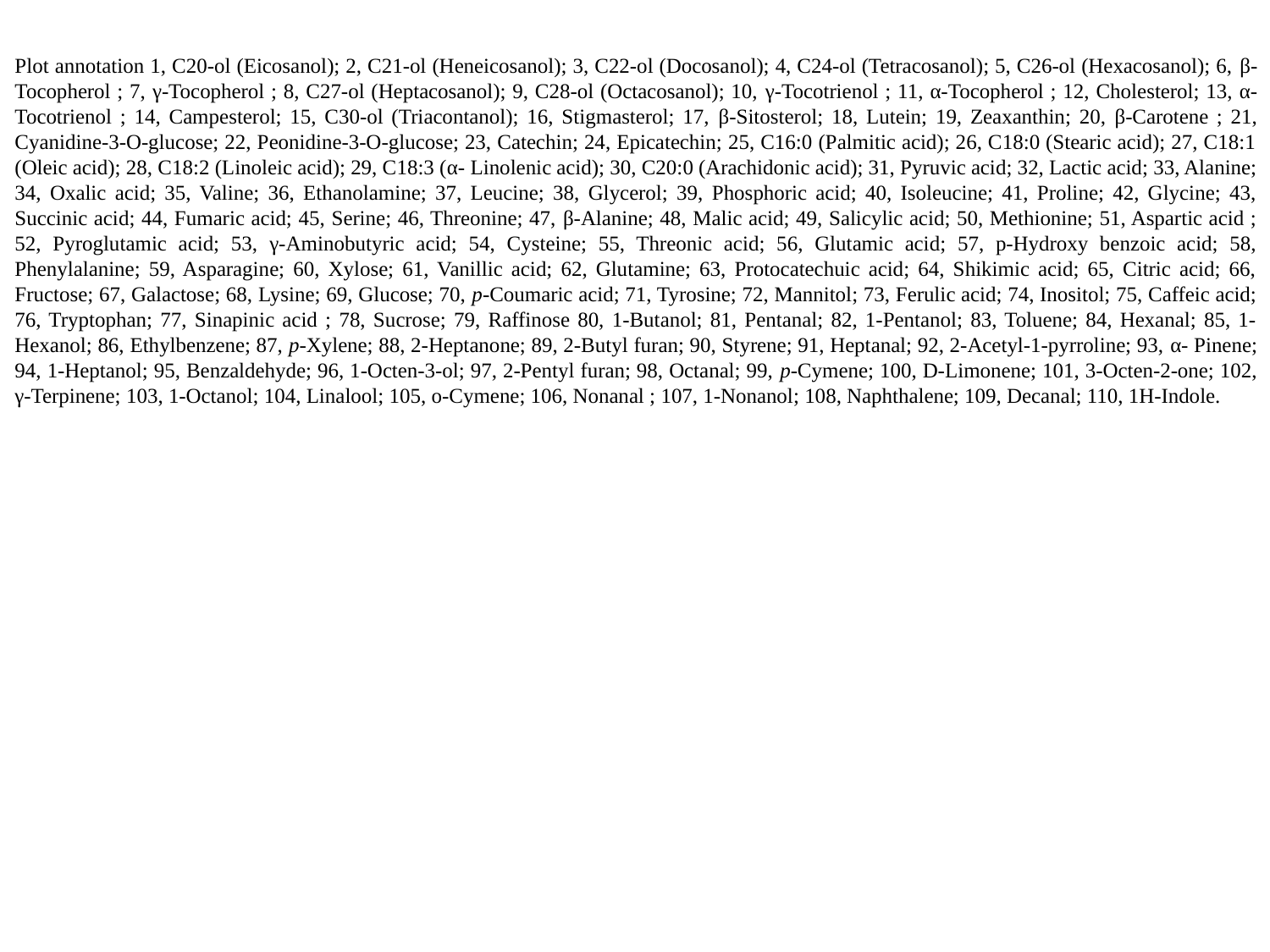

Plot annotation 1, C20-ol (Eicosanol); 2, C21-ol (Heneicosanol); 3, C22-ol (Docosanol); 4, C24-ol (Tetracosanol); 5, C26-ol (Hexacosanol); 6, β-Tocopherol ; 7, γ-Tocopherol ; 8, C27-ol (Heptacosanol); 9, C28-ol (Octacosanol); 10, γ-Tocotrienol ; 11, α-Tocopherol ; 12, Cholesterol; 13, α-Tocotrienol ; 14, Campesterol; 15, C30-ol (Triacontanol); 16, Stigmasterol; 17, β-Sitosterol; 18, Lutein; 19, Zeaxanthin; 20, β-Carotene ; 21, Cyanidine-3-O-glucose; 22, Peonidine-3-O-glucose; 23, Catechin; 24, Epicatechin; 25, C16:0 (Palmitic acid); 26, C18:0 (Stearic acid); 27, C18:1 (Oleic acid); 28, C18:2 (Linoleic acid); 29, C18:3 (α- Linolenic acid); 30, C20:0 (Arachidonic acid); 31, Pyruvic acid; 32, Lactic acid; 33, Alanine; 34, Oxalic acid; 35, Valine; 36, Ethanolamine; 37, Leucine; 38, Glycerol; 39, Phosphoric acid; 40, Isoleucine; 41, Proline; 42, Glycine; 43, Succinic acid; 44, Fumaric acid; 45, Serine; 46, Threonine; 47, β-Alanine; 48, Malic acid; 49, Salicylic acid; 50, Methionine; 51, Aspartic acid ; 52, Pyroglutamic acid; 53, γ-Aminobutyric acid; 54, Cysteine; 55, Threonic acid; 56, Glutamic acid; 57, p-Hydroxy benzoic acid; 58, Phenylalanine; 59, Asparagine; 60, Xylose; 61, Vanillic acid; 62, Glutamine; 63, Protocatechuic acid; 64, Shikimic acid; 65, Citric acid; 66, Fructose; 67, Galactose; 68, Lysine; 69, Glucose; 70, p-Coumaric acid; 71, Tyrosine; 72, Mannitol; 73, Ferulic acid; 74, Inositol; 75, Caffeic acid; 76, Tryptophan; 77, Sinapinic acid ; 78, Sucrose; 79, Raffinose 80, 1-Butanol; 81, Pentanal; 82, 1-Pentanol; 83, Toluene; 84, Hexanal; 85, 1-Hexanol; 86, Ethylbenzene; 87, p-Xylene; 88, 2-Heptanone; 89, 2-Butyl furan; 90, Styrene; 91, Heptanal; 92, 2-Acetyl-1-pyrroline; 93, α- Pinene; 94, 1-Heptanol; 95, Benzaldehyde; 96, 1-Octen-3-ol; 97, 2-Pentyl furan; 98, Octanal; 99, p-Cymene; 100, D-Limonene; 101, 3-Octen-2-one; 102, γ-Terpinene; 103, 1-Octanol; 104, Linalool; 105, o-Cymene; 106, Nonanal ; 107, 1-Nonanol; 108, Naphthalene; 109, Decanal; 110, 1H-Indole.

## Slide 5
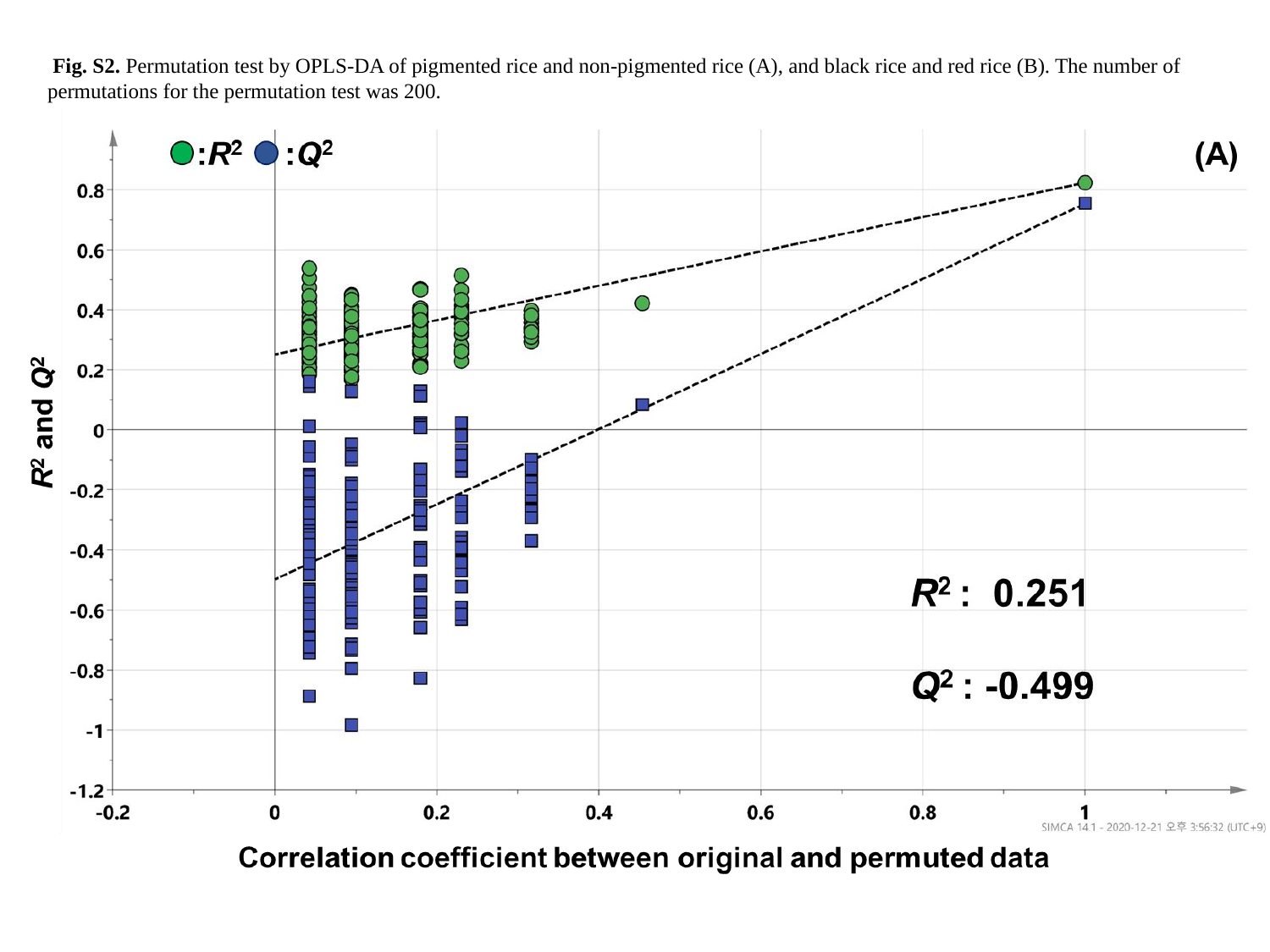

Fig. S2. Permutation test by OPLS-DA of pigmented rice and non-pigmented rice (A), and black rice and red rice (B). The number of permutations for the permutation test was 200.

## Slide 6
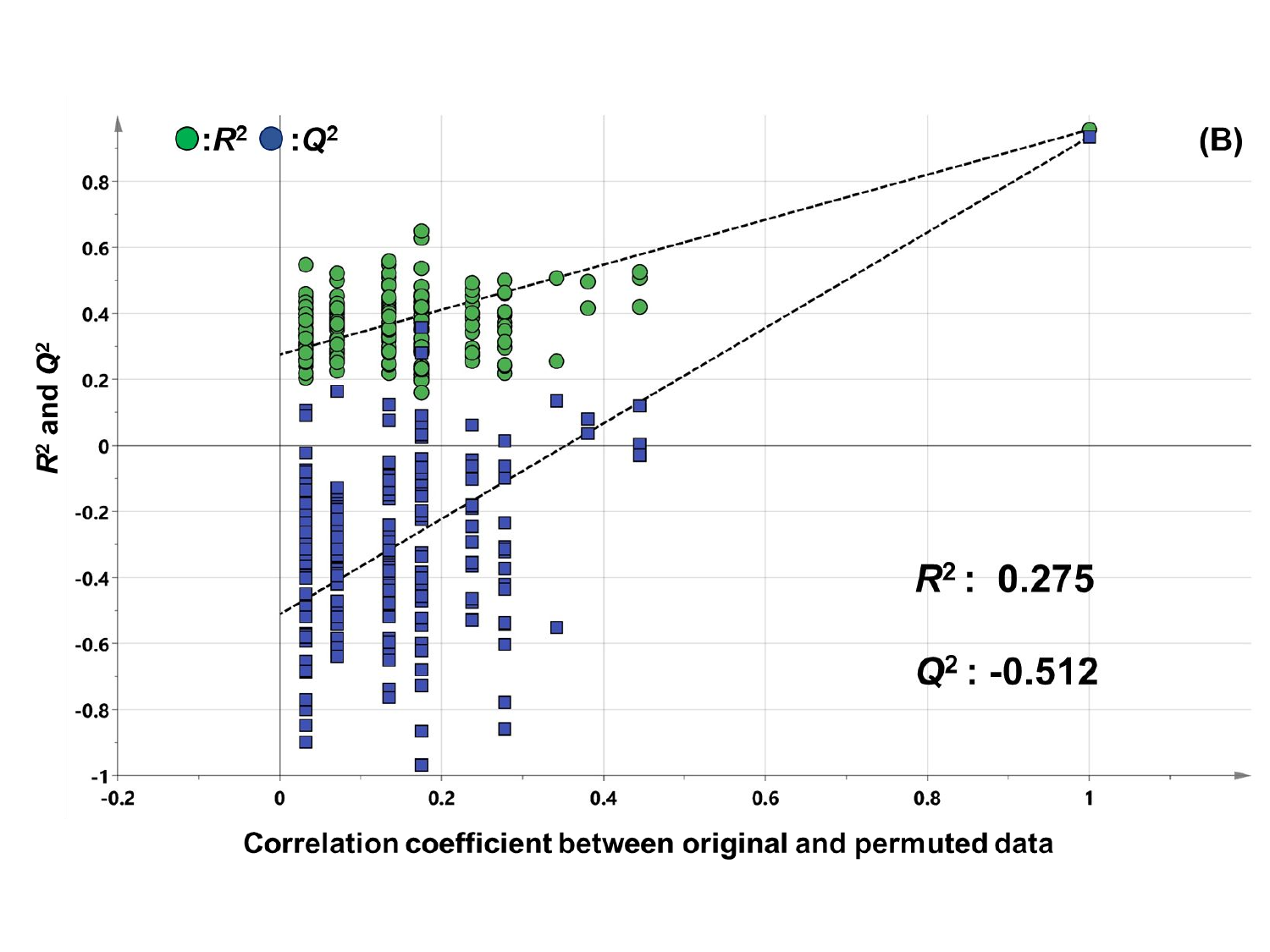

## Slide 7
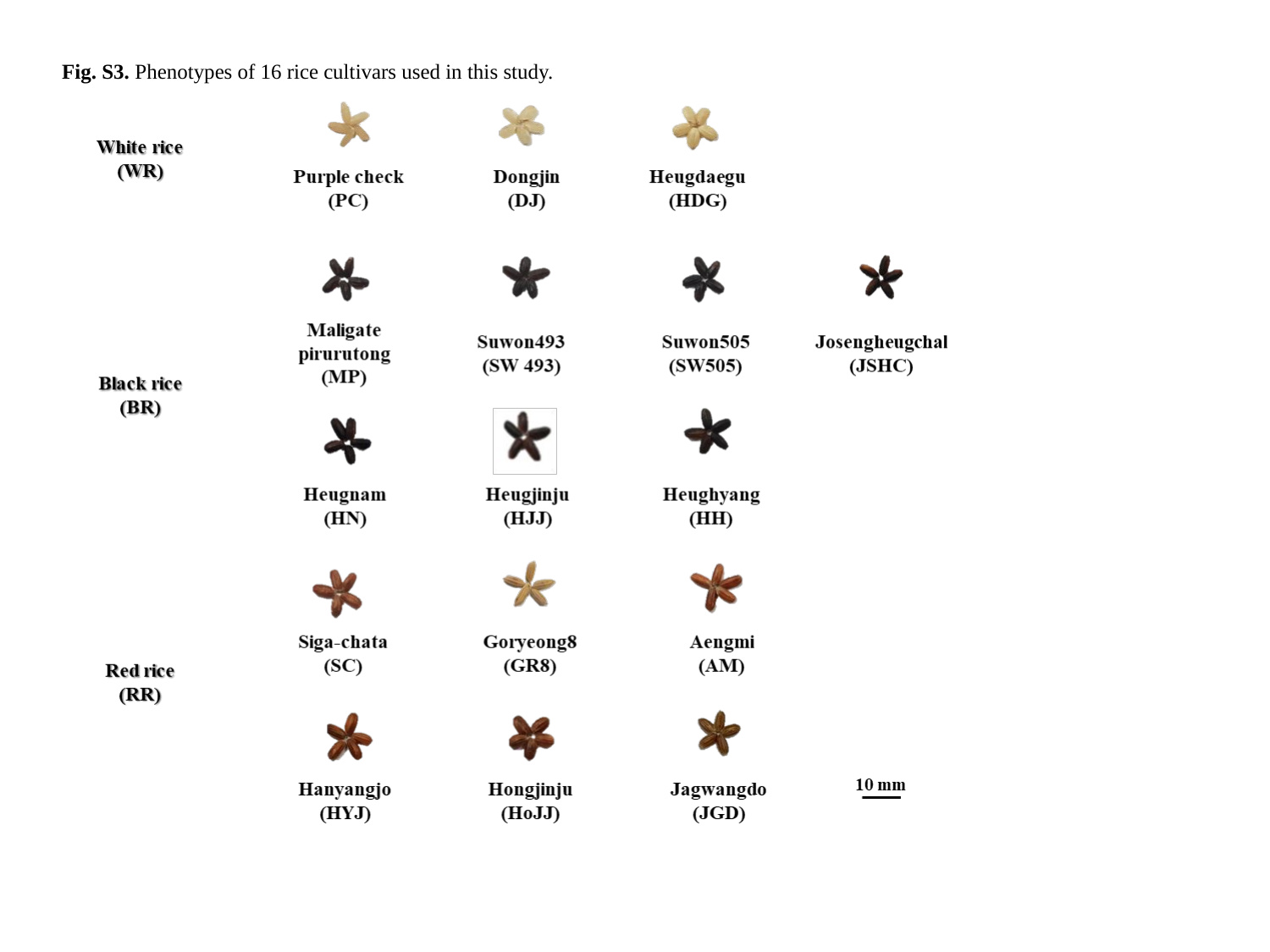

Fig. S3. Phenotypes of 16 rice cultivars used in this study.

## Slide 8
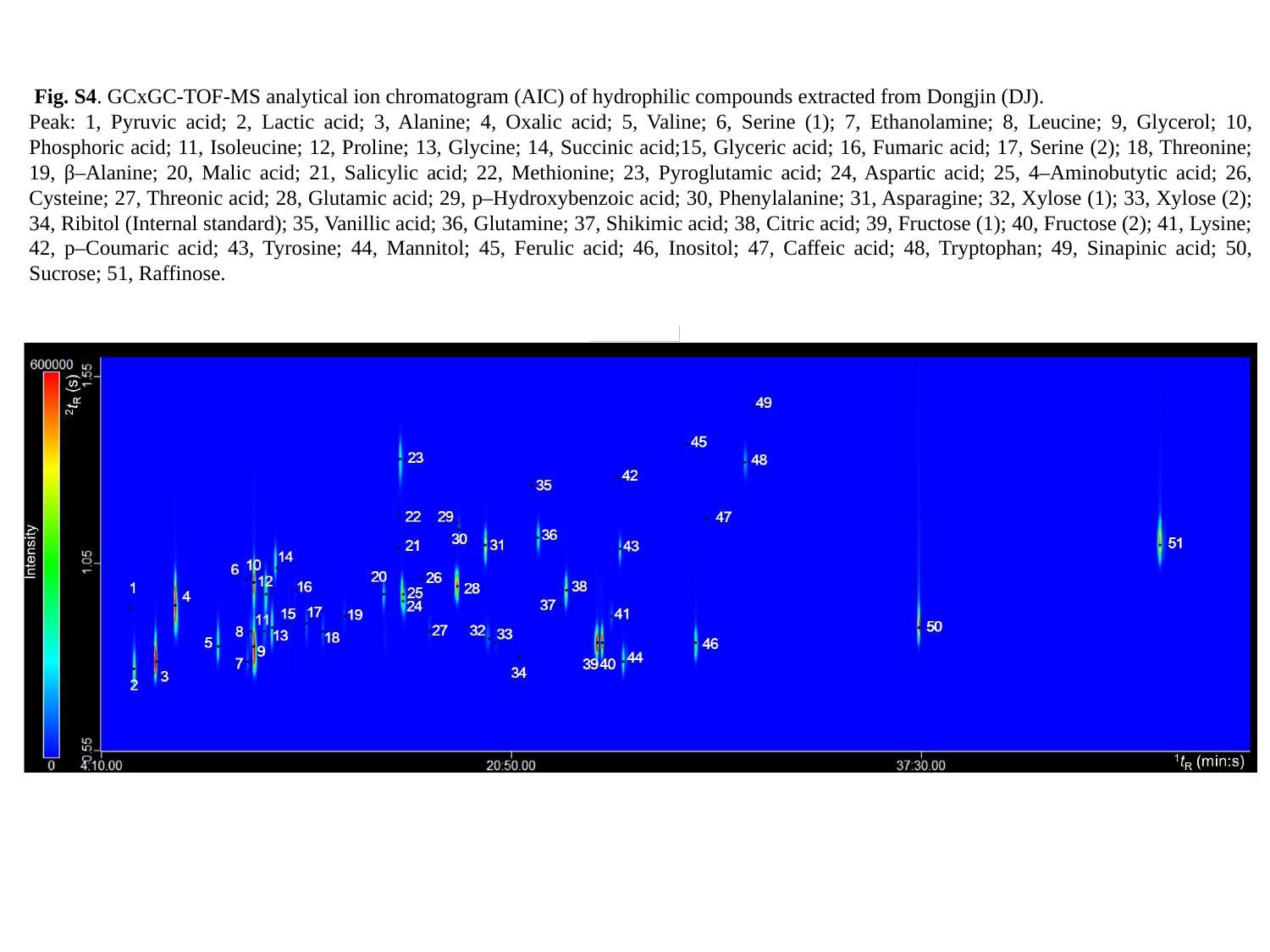

Fig. S4. GCxGC-TOF-MS analytical ion chromatogram (AIC) of hydrophilic compounds extracted from Dongjin (DJ).
Peak: 1, Pyruvic acid; 2, Lactic acid; 3, Alanine; 4, Oxalic acid; 5, Valine; 6, Serine (1); 7, Ethanolamine; 8, Leucine; 9, Glycerol; 10, Phosphoric acid; 11, Isoleucine; 12, Proline; 13, Glycine; 14, Succinic acid;15, Glyceric acid; 16, Fumaric acid; 17, Serine (2); 18, Threonine; 19, β–Alanine; 20, Malic acid; 21, Salicylic acid; 22, Methionine; 23, Pyroglutamic acid; 24, Aspartic acid; 25, 4–Aminobutytic acid; 26, Cysteine; 27, Threonic acid; 28, Glutamic acid; 29, p–Hydroxybenzoic acid; 30, Phenylalanine; 31, Asparagine; 32, Xylose (1); 33, Xylose (2); 34, Ribitol (Internal standard); 35, Vanillic acid; 36, Glutamine; 37, Shikimic acid; 38, Citric acid; 39, Fructose (1); 40, Fructose (2); 41, Lysine; 42, p–Coumaric acid; 43, Tyrosine; 44, Mannitol; 45, Ferulic acid; 46, Inositol; 47, Caffeic acid; 48, Tryptophan; 49, Sinapinic acid; 50, Sucrose; 51, Raffinose.

## Slide 9
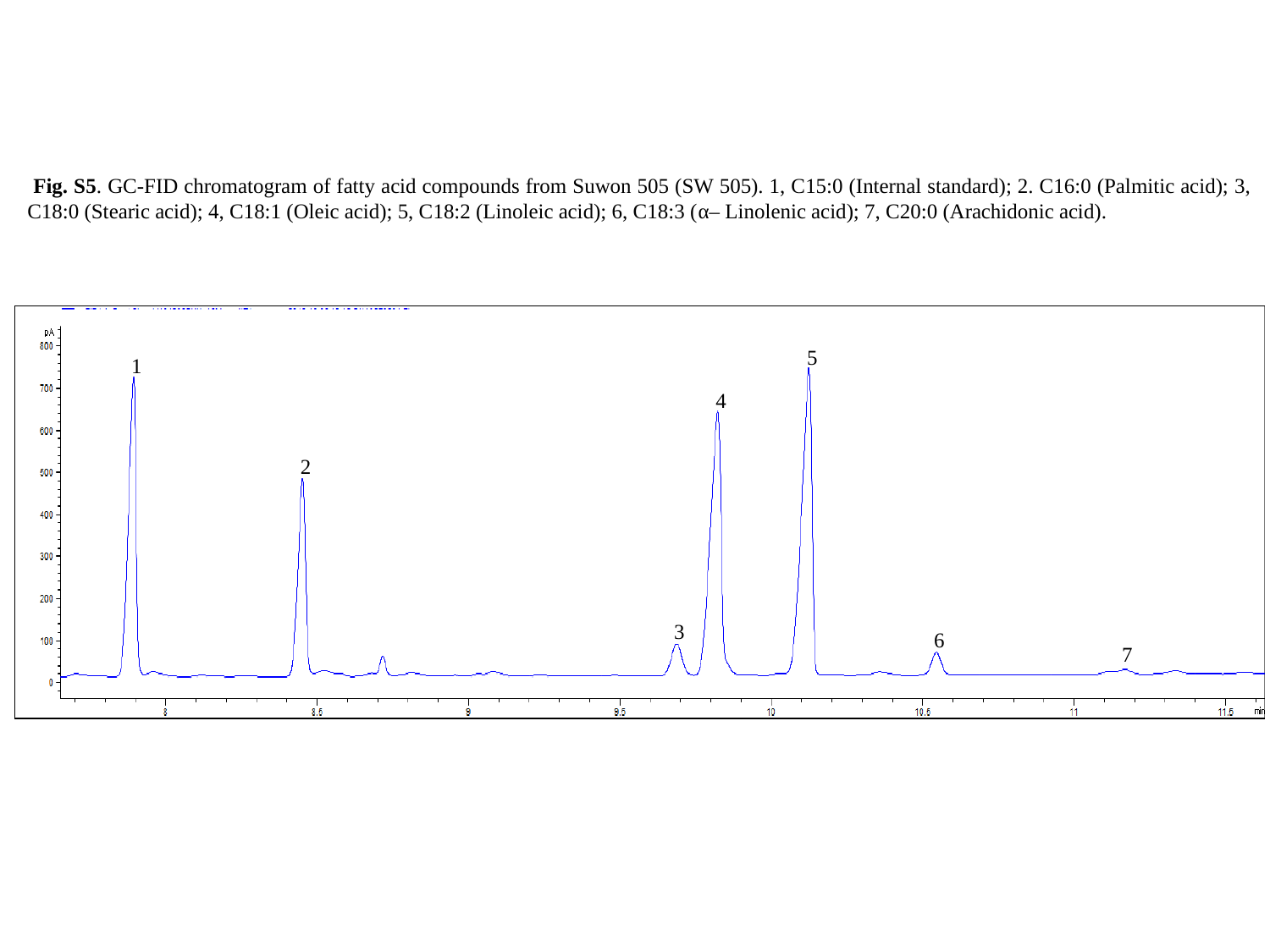

Fig. S5. GC-FID chromatogram of fatty acid compounds from Suwon 505 (SW 505). 1, C15:0 (Internal standard); 2. C16:0 (Palmitic acid); 3, C18:0 (Stearic acid); 4, C18:1 (Oleic acid); 5, C18:2 (Linoleic acid); 6, C18:3 (α– Linolenic acid); 7, C20:0 (Arachidonic acid).
5
1
4
2
3
6
7

## Slide 10
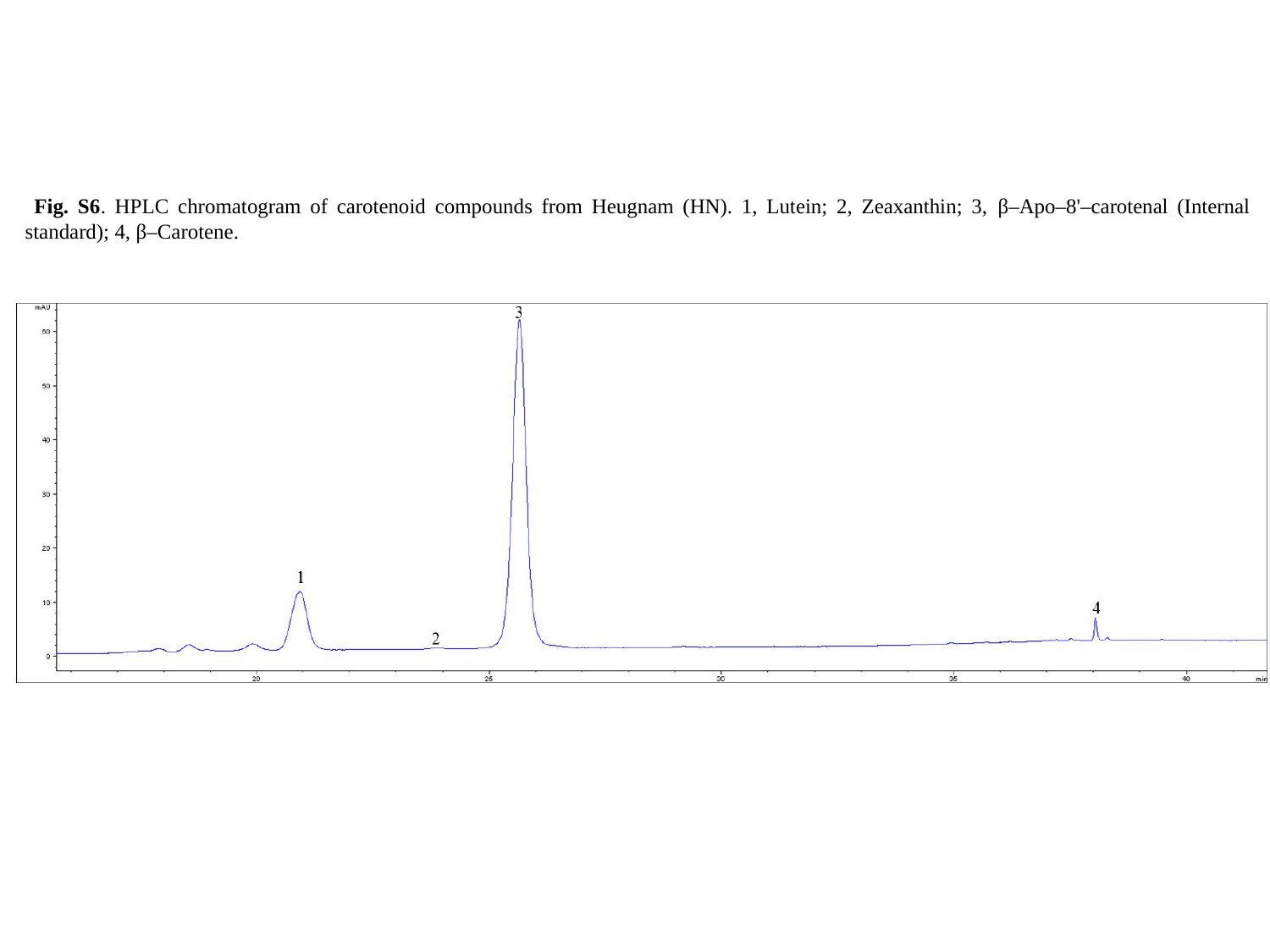

Fig. S6. HPLC chromatogram of carotenoid compounds from Heugnam (HN). 1, Lutein; 2, Zeaxanthin; 3, β–Apo–8'–carotenal (Internal standard); 4, β–Carotene.

## Slide 11
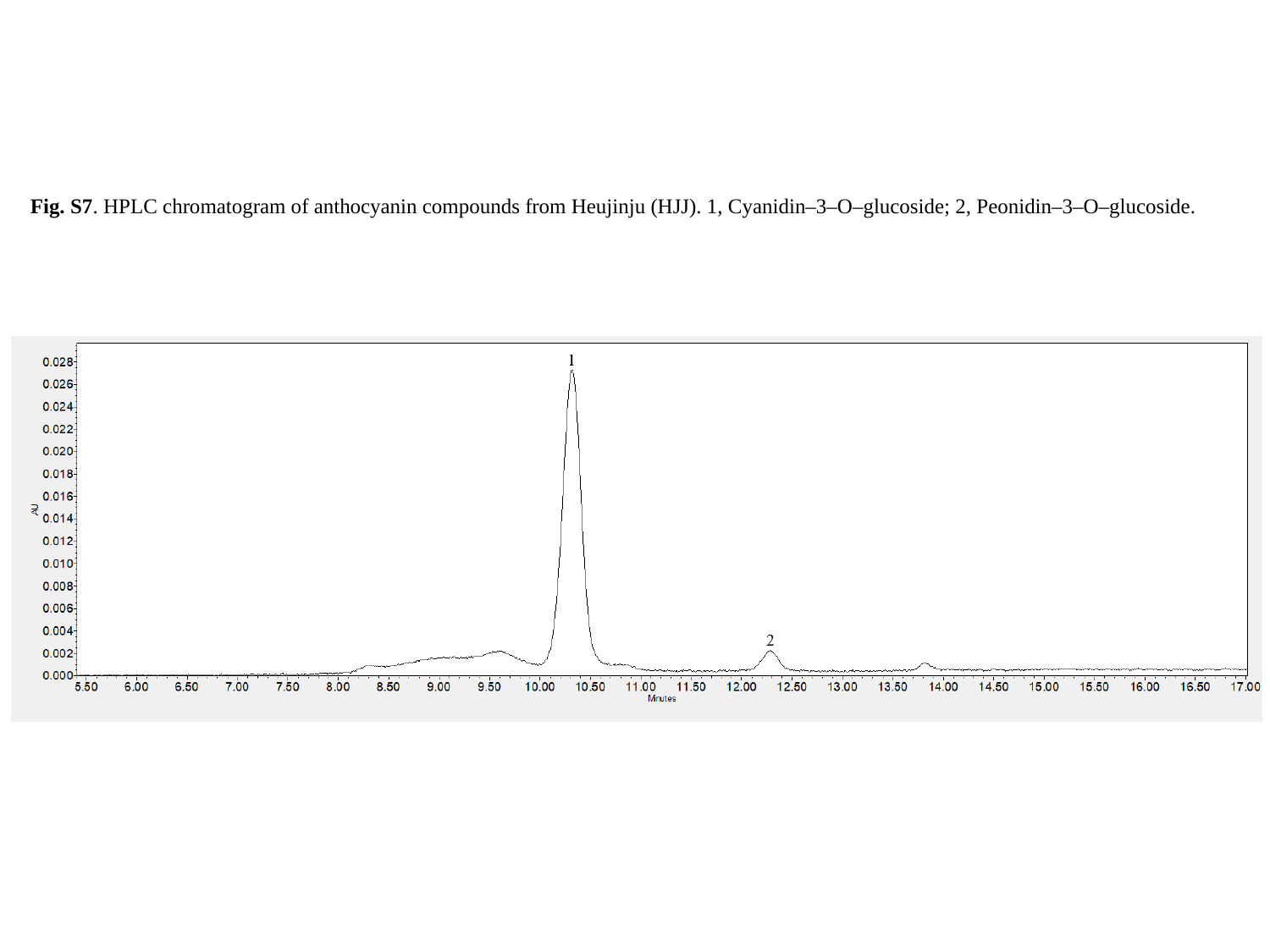

Fig. S7. HPLC chromatogram of anthocyanin compounds from Heujinju (HJJ). 1, Cyanidin–3–O–glucoside; 2, Peonidin–3–O–glucoside.

## Slide 12
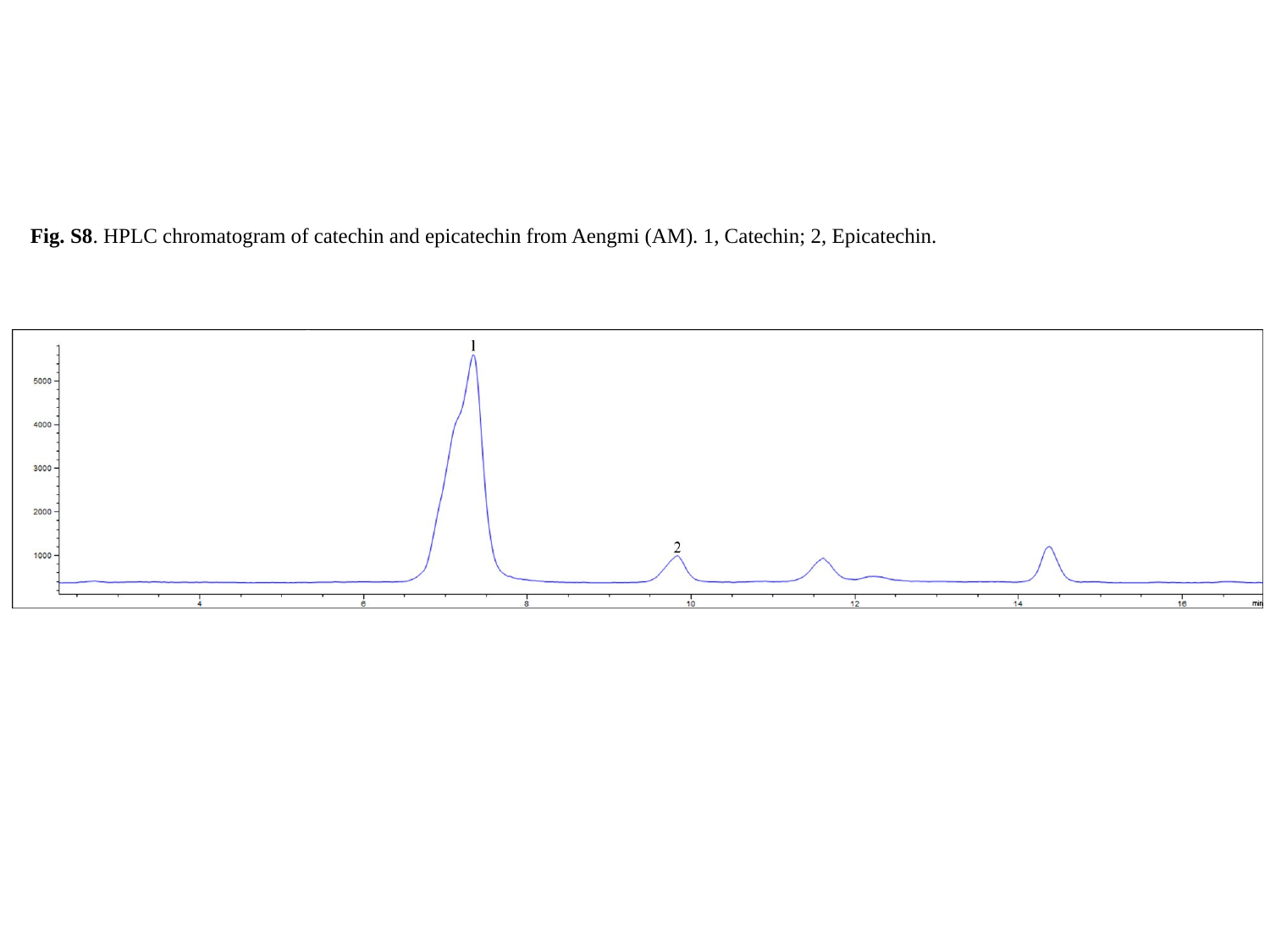

Fig. S8. HPLC chromatogram of catechin and epicatechin from Aengmi (AM). 1, Catechin; 2, Epicatechin.

## Slide 13
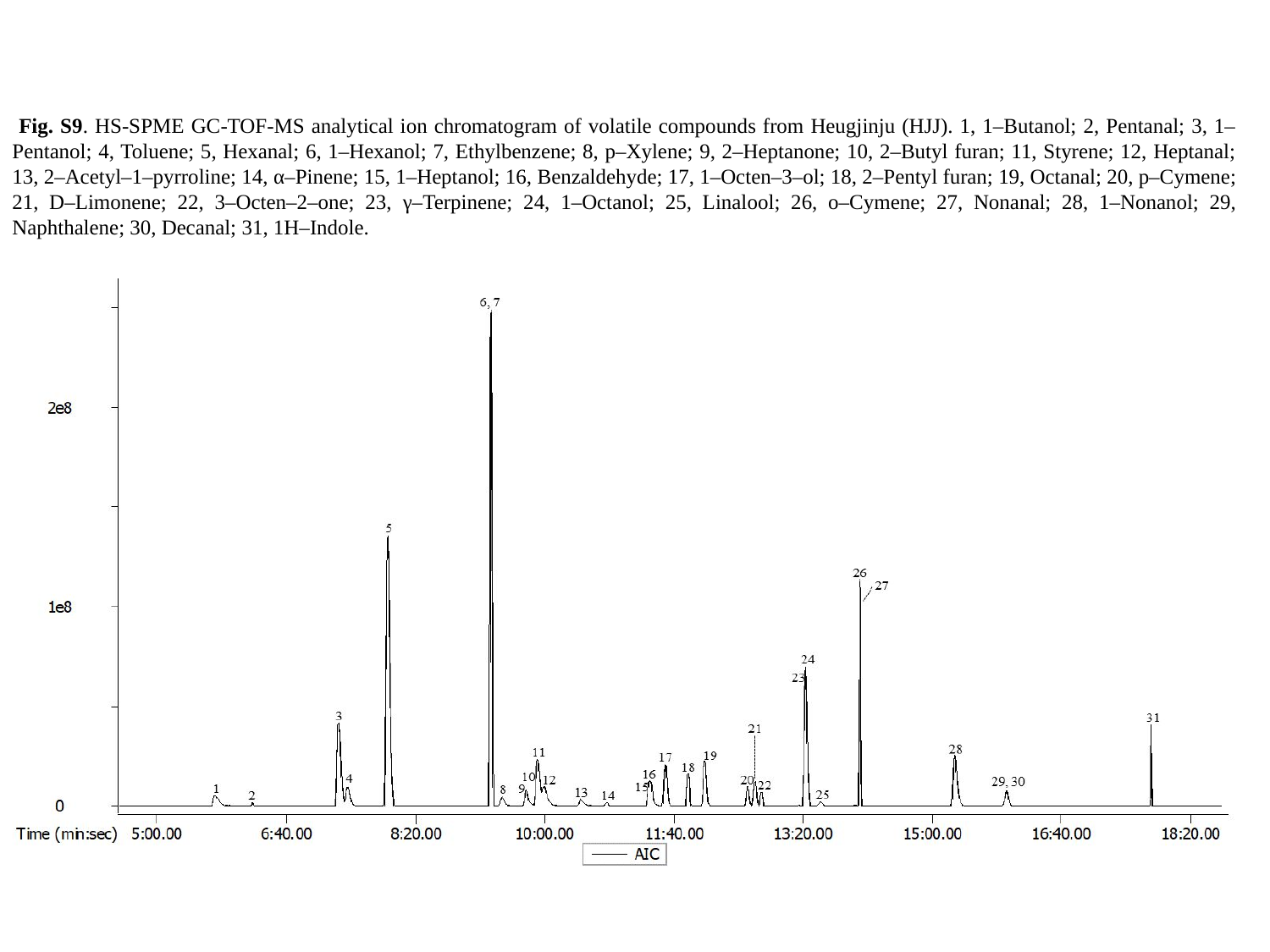

Fig. S9. HS-SPME GC-TOF-MS analytical ion chromatogram of volatile compounds from Heugjinju (HJJ). 1, 1–Butanol; 2, Pentanal; 3, 1–Pentanol; 4, Toluene; 5, Hexanal; 6, 1–Hexanol; 7, Ethylbenzene; 8, p–Xylene; 9, 2–Heptanone; 10, 2–Butyl furan; 11, Styrene; 12, Heptanal; 13, 2–Acetyl–1–pyrroline; 14, α–Pinene; 15, 1–Heptanol; 16, Benzaldehyde; 17, 1–Octen–3–ol; 18, 2–Pentyl furan; 19, Octanal; 20, p–Cymene; 21, D–Limonene; 22, 3–Octen–2–one; 23, γ–Terpinene; 24, 1–Octanol; 25, Linalool; 26, o–Cymene; 27, Nonanal; 28, 1–Nonanol; 29, Naphthalene; 30, Decanal; 31, 1H–Indole.
